# Varicella zoster virus glycoprotein C increases chemokine-mediated leukocyte migration

**DOI:** 10.1371/journal.ppat.1006346

**Published:** 2017-05-25

**Authors:** Víctor González-Motos, Carina Jürgens, Birgit Ritter, Kai A. Kropp, Verónica Durán, Olav Larsen, Anne Binz, Werner J. D. Ouwendijk, Tihana Lenac Rovis, Stipan Jonjic, Georges M. G. M. Verjans, Beate Sodeik, Thomas Krey, Rudolf Bauerfeind, Thomas F. Schulz, Benedikt B. Kaufer, Ulrich Kalinke, Amanda E. I. Proudfoot, Mette M. Rosenkilde, Abel Viejo-Borbolla

**Affiliations:** 1 Institute of Virology, Hannover Medical School, Hannover, Germany; 2 University of Veterinary Medicine Hannover, Foundation, Hannover, Germany; 3 Institute for Experimental Infection Research, TWINCORE, Centre for Experimental and Clinical Infection Research, a joint venture between the Hannover Medical School and the Helmholtz Centre for Infection Research, Hannover, Germany; 4 Department of Biomedical Sciences, Faculty of Health and Medical Science, University of Copenhagen, Copenhagen, Denmark; 5 Department of Viroscience, Erasmus MC, Rotterdam, The Netherlands; 6 Center for Proteomics and Department of Histology and Embryology, Faculty of Medicine, University of Rijeka, Rijeka, Croatia; 7 Research Center for Emerging Infections and Zoonoses, University of Veterinary Medicine Hannover, Hannover, Germany; 8 German Center for Infection Research (DZIF), Hannover-Braunschweig, Germany; 9 Research Core Unit for Laser Microscopy, Hannover Medical School, Hannover, Germany; 10 Institute for Virology, Freie Universität Berlin, Berlin, Germany; 11 NovImmune, Geneva, Switzerland; NIH/NIAID, UNITED STATES

## Abstract

Varicella zoster virus (VZV) is a highly prevalent human pathogen that establishes latency in neurons of the peripheral nervous system. Primary infection causes varicella whereas reactivation results in zoster, which is often followed by chronic pain in adults. Following infection of epithelial cells in the respiratory tract, VZV spreads within the host by hijacking leukocytes, including T cells, in the tonsils and other regional lymph nodes, and modifying their activity. In spite of its importance in pathogenesis, the mechanism of dissemination remains poorly understood. Here we addressed the influence of VZV on leukocyte migration and found that the purified recombinant soluble ectodomain of VZV glycoprotein C (rSgC) binds chemokines with high affinity. Functional experiments show that VZV rSgC potentiates chemokine activity, enhancing the migration of monocyte and T cell lines and, most importantly, human tonsillar leukocytes at low chemokine concentrations. Binding and potentiation of chemokine activity occurs through the C-terminal part of gC ectodomain, containing predicted immunoglobulin-like domains. The mechanism of action of VZV rSgC requires interaction with the chemokine and signalling through the chemokine receptor. Finally, we show that VZV viral particles enhance chemokine-dependent T cell migration and that gC is partially required for this activity. We propose that VZV gC activity facilitates the recruitment and subsequent infection of leukocytes and thereby enhances VZV systemic dissemination in humans.

## Author summary

Varicella zoster virus (VZV) causes two main pathologies in humans, chickenpox during primary infection, and shingles following reactivation. The latter is a painful condition that is often followed by chronic pain in a large numbers of shingles patients. Despite the existence of a vaccine, shingles-related complications cause expenses of more than $1 billion per year in the USA alone. Following primary infection, the virus infects leukocytes including T cells, spreading to the skin causing chickenpox. Direct infection of neurons from leukocytes has also been postulated. Given the relevance of leukocytes in VZV biology and the importance of chemokines in directing their migration, we investigated whether VZV modulates the function of chemokines. Our results show that VZV glycoprotein C potentiates the activity of chemokines, inducing higher migration of human leukocytes at low chemokine concentration. This may attract additional susceptible leukocytes to the site of infection enhancing virus spread and pathogenesis.

## Introduction

Varicella zoster virus (VZV) belongs to the *Alphaherpesvirinae* subfamily and establishes latency in ganglia of the peripheral nervous system [1]. VZV causes varicella during primary infection and zoster, a painful vesicular rash, following reactivation. There are licensed vaccines to prevent varicella and zoster. However, the annual incidence of zoster increases with age, being approximately 0.7–1% in individuals older than 65 years old in the USA and Europe [2–5]. Zoster is frequently followed by post-herpetic neuralgia (PHN), the second most common type of neuropathic pain worldwide, in the elderly [3, 6–8]. Zoster and PHN related complications are associated with high health care costs [9, 10]. The cellular and viral factors involved in the induction of pain by VZV are not fully known. This is in part due to the host specificity of VZV that highly restricts the use of animal models to study VZV pathogenesis *in vivo*.

During the natural course of infection VZV infects epithelial cells in the mucosa of the respiratory tract. Subsequently it infects leukocytes including dendritic and T cells [11–16], most likely in the proximity of the Waldeyer’s tonsillar ring, allowing dissemination of the virus to internal organs, the skin and sensory ganglia [11, 12, 15, 17–19]. VZV infection modulates gene expression of T cells, inducing a phenotype associated with leukocyte migration towards skin where infection of keratinocytes and free nerve endings occurs [20]. VZV establishes latency in neurons either following retrograde transport from the skin or through direct transmission from infected leukocytes [21–23]. All these data point to the relevance of leukocytes in VZV dissemination and to the ability of VZV to modulate T cell activity, including migration. Whether VZV recruits leukocytes to facilitate spread remains unknown.

Leukocyte migration is a highly regulated process [24] with chemokines playing an essential role [25]. There are four classes of chemokines classified according to the relative position of the N-terminal cysteine residues into CXC, CC, C and CX3C [26]. To function *in vivo*, chemokines interact with glycosaminoglycans (GAGs) [27, 28] and G protein-coupled receptors (GPCRs). Binding of the chemokine to GPCRs activates Gαi proteins, triggering a series of signalling events that culminate in leukocyte transmigration to the infected tissue [29]. Several viruses modulate leukocyte migration through the regulation of chemokine activity. Some members of the *Pox-* and *Herpesviridae* families express chemokine binding GPCRs [30], while others express secreted or type I transmembrane proteins that bind chemokines with high affinity termed viral chemokine binding proteins (vCKBP) [31]. The vCKBP have low or no sequence identity between themselves or with host proteins. The majority of the described vCKBP inhibit chemokine activity, through impairing the interaction of the chemokine with the GPCR, GAGs or both [31, 32]. The exception to this rule is soluble glycoprotein G (SgG) from herpes simplex virus type 1 and 2 (HSV-1 and HSV-2, respectively), which, in contrast to gG from animal alphaherpesviruses [33], enhances chemokine-mediated migration [34]. So far no chemokine binding activity has been described for VZV, which lacks the orthologous gG gene (*US4*) [35, 36].

Due to the relevance of leukocyte migration in VZV spread and subsequent pathogenesis we investigated the possible modulation of leukocyte migration by VZV. Moreover, chemokines and leukocytes are also involved in the generation and chronicity of pain [37, 38], a feature of VZV pathology. We focused on VZV glycoprotein C (gC) encoded by open reading frame 14 (ORF14) [39] since several results point to a potential role for gC in VZV pathogenesis: (i) VZV isolates lacking gC expression replicate at lower levels than the parental virus in human skin implants in severe combined immunodeficiency mice and in human foetal skin organ culture [40, 41]; (ii) VZV gC is not essential for replication *in vitro* [42] and passage of VZV in culture can result in loss of gC expression [40]; (iii) the attenuated vaccine strain vOka expresses lower levels of gC than parental pOka or other wild type strains [39, 43]. VZV gC is a type I transmembrane protein of unknown function. Furthermore, it is unclear if gC or a particular gC domain is secreted by infected cells by proteolytic cleavage or due to alternative splicing as reported for HSV-1 gC [44].

Our results show that recombinant soluble VZV gC ectodomain (rSgC) binds chemokines and potentiates chemokine-dependent leukocyte migration, including that of human tonsillar leukocytes, the target of VZV during primary infection. The interaction with chemokines is of high affinity and takes place through the C-terminal part of gC ectodomain containing two predicted immunoglobulin-like domains (IgD). This region is also sufficient for potentiation of chemokine activity. Moreover, we show that VZV rSgC binds to the cell surface via a specific interaction with GAGs taking place through an N-terminal repeated domain. Interaction of rSgC with the cell surface through GAGs is not required for potentiation of chemokine activity *in vitro*. However, binding to the chemokine and signalling through its receptor are required for rSgC activity. Finally, cell-free VZV enhances chemokine-dependent T cell migration and deletion of gC reduces this enhancement. We propose that VZV gC activity increases the chemokine-mediated attraction of leukocytes to the site of infection improving dissemination of the virus within the host.

## Results

### Novel vCKBP VZV rSgC binds chemokines with high affinity

**Fig 1:**
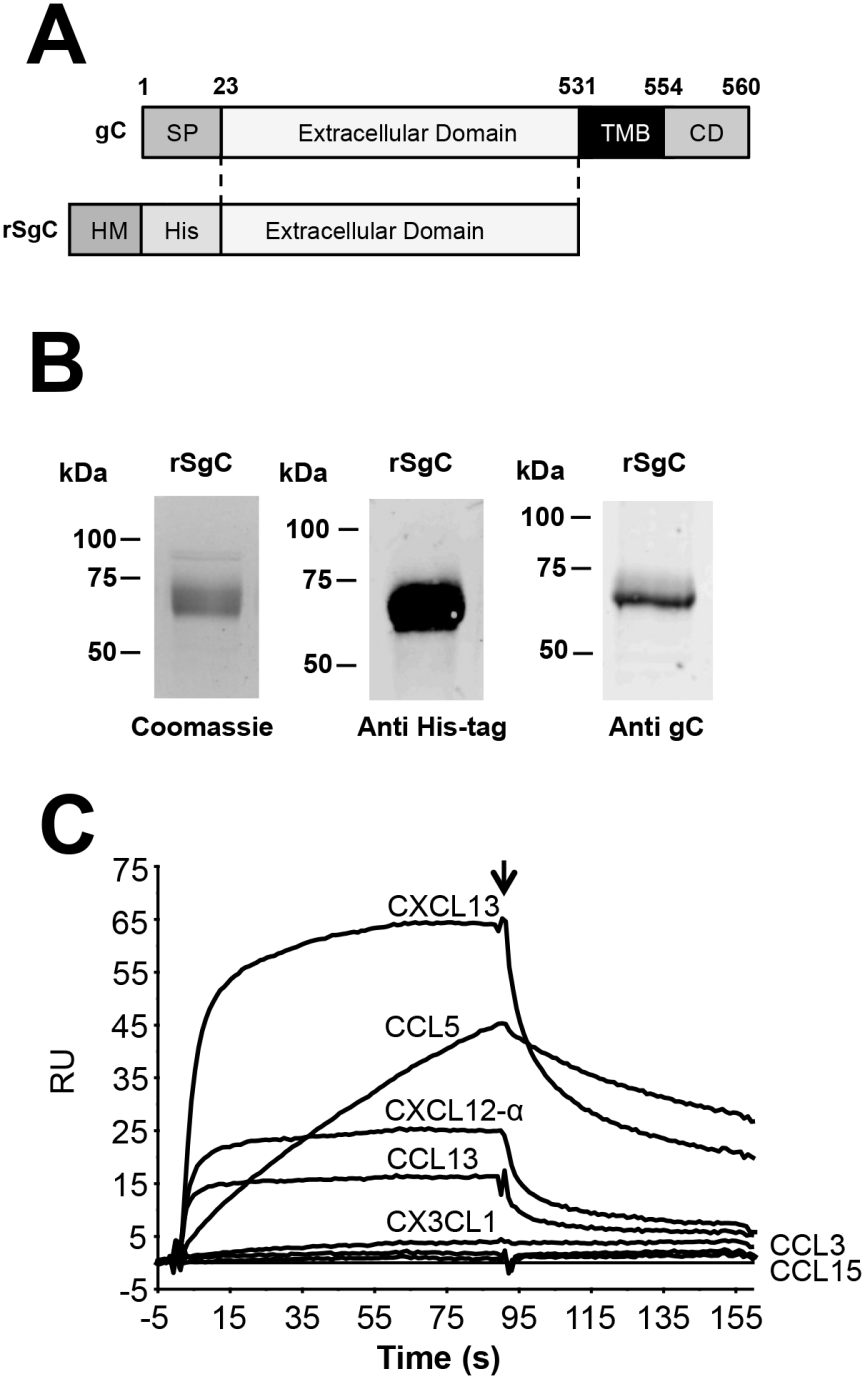
Determination of VZV rSgC chemokine binding properties. (**A**) Schematic representation of full-length VZV gC protein (top) and the derived construct to express recombinant soluble VZV gC ectodomain (rSgC, bottom) in Hi-5 insect cells. Numbers indicate amino acid positions within VZV gC Dumas strain. The VZV gC signal peptide (SP) was substituted by that of the honey bee melittin (HM) to improve secretion in insect cells. A histidine tag (His) was introduced at the N-terminus to facilitate purification of rSgC by affinity chromatography. (**B**) Purified rSgC was detected by Coomassie staining (left panel) or by Western blotting using antibodies to the His-tag (middle panel) or to VZV gC (right panel). (**C**) Sensorgram showing association and dissociation phases of the interaction between rSgC and selected chemokines injected at a concentration of 100 nM. The arrow indicates the end of the chemokine injection. Positive (CXCL13, CXCL12-α, CCL5, CCL13) and negative (CCL3, CCL15 and CX3CL1) interactions are shown. Abbreviations: RU, resonance units. kDa, kiloDaltons; TMB, transmembrane; CD, cytoplasmic domain.

In order to address whether VZV gC can modulate leukocyte migration we expressed a recombinant, soluble VZV gC ectodomain (VZV rSgC, amino acids 23–531 in gC from Dumas strain [36]) containing an N-terminal histidine tag (His-tag) in insect cells using the baculovirus expression system (Fig 1A). To enhance protein secretion in insect cells we substituted the predicted VZV gC signal peptide by that of the honey bee melittin. The protein was purified by affinity chromatography and detected by Coomassie staining and western blotting using anti His-tag and anti VZV gC [45] monoclonal antibodies (Fig 1B). We immobilised purified VZV rSgC on a CM4 Biacore chip and performed a binding screening with 43 human chemokines (see Materials and methods) injected at a concentration of 100 nM using a 30 μl/min flow rate to reduce mass transfer. A sensorgram showing selected curves from this assay is shown in Fig 1C. Surface plasmon resonance (SPR) data showed that VZV rSgC bound a broad range of human CXC and CC chemokines: CXCL1, CXCL2, CXCL4, CXCL6, CXCL9, CXCL10, CXCL11, CXCL12-α, CXCL12-β, CXCL13, CXCL14, CCL1, CCL5, CCL11, CCL13, CCL16, CCL17, CCL18, CCL19, CCL20, CCL21, CCL22, CCL25, CCL26, CCL27 and CCL28. The affinity of the interaction was high, in the nanomolar range (Table 1). Weak interactors, according to the response obtained in the SPR experiments, were CXCL16, CCL2, CCL7, CCL8, and CCL27. VZV rSgC did not interact with CCL3, CCL4, CCL14, CCL15, CCL23, CXCL3, CXCL5, CXCL7, CXCL8, CX3CL1 and XCL1. Interaction with a high number of chemokines has been previously shown for other vCKBP such as murine gammaherpesvirus 68 (MHV-68) M3 [46, 47]. M3 specificity is broader than that of rSgC, since it can also interact with members of the CX3CL1 and XCL1 families. The different association and dissociation rates suggest different types of binding properties between rSgC and distinct chemokines. Overall, our results show that VZV gC is a vCKBP that interacts with high affinity with a broad range, but not all, of human chemokines belonging to the CXC and CC subfamilies.

**Table 1:** Binding parameters of different chemokines to rSgC. N.D: Not determined.

Chemokine	Binding	Ka (1/Ms)	Kd (1/s)	KD (M)	t(1/2)s
hCXCL12-α	Yes	1.60 x 10^7^	0.004293	2.69 x 10^−10^	161.4256
hCXCL13	Yes	5.94 x 10^6^	0.002506	4.22 x 10^−10^	276.5363
hCCL2	Yes	1.73 x 10^5^	0.001012	5.84 x 10^−9^	684.7826
hCCL13	Yes	1.04 x 10^7^	0.001836	1.76 x 10^−10^	377.451
hCCL19	Yes	1.54 x 10^7^	0.002117	1.38 x 10^−10^	327.35
hCCL3	No	N.D.	N.D.	N.D.	N.D.
hCCL15	No	N.D.	N.D.	N.D.	N.D.

### VZV rSgC enhances chemokine-dependent leukocyte migration of human tonsillar cells

**Fig 2:**
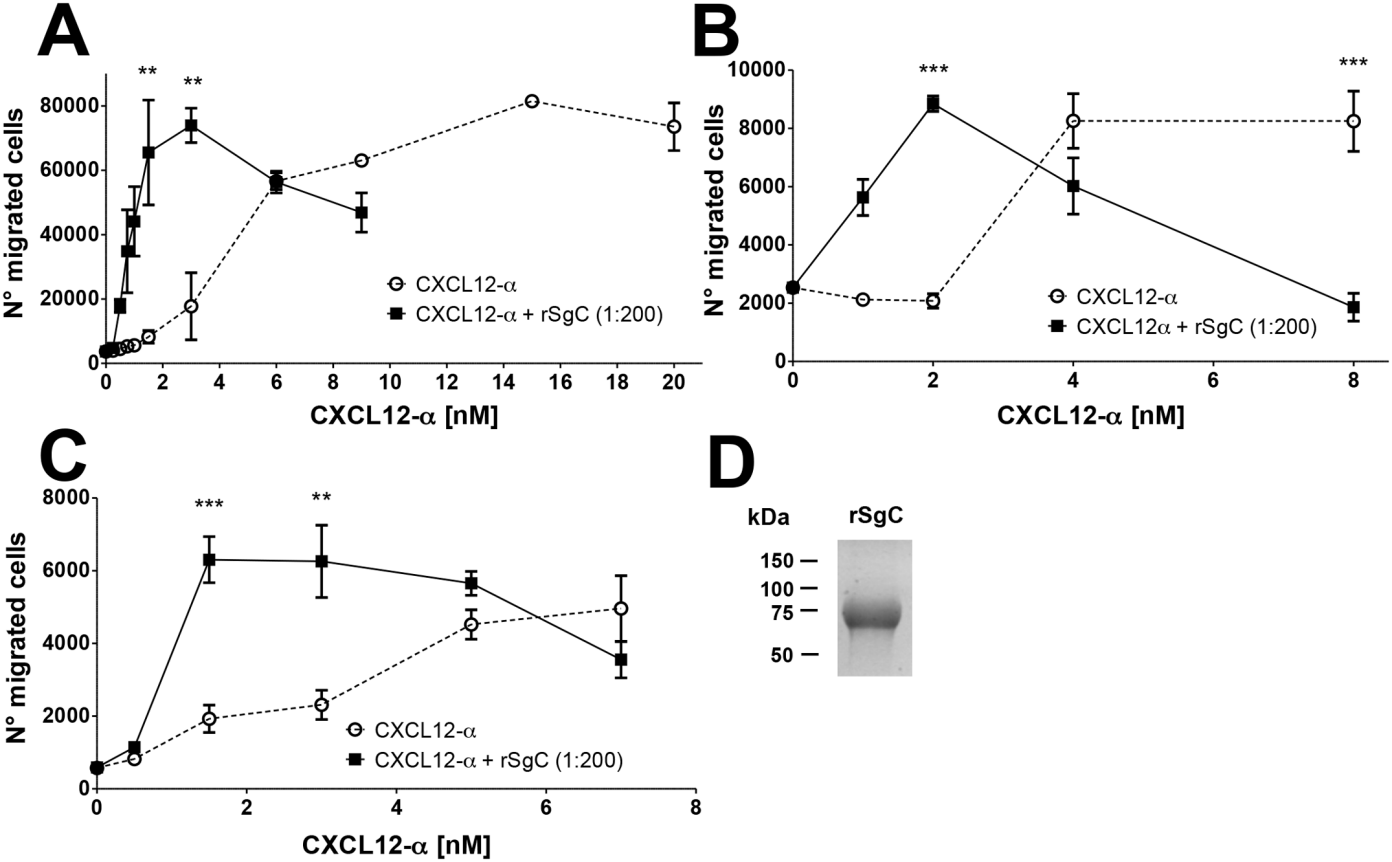
VZV rSgC enhances chemokine-dependent migration. Chemotaxis of Jurkat (**A**) and MonoMac-1 (**B**) cell lines and human primary tonsillar leukocytes (**C**) towards increasing concentrations of CXCL12-α alone or in the presence of a 1:200 molar ratio of chemokine:rSgC. In all experiments the chemokine was incubated with or without VZV rSgC at 37°C in a humidified incubator prior to the addition of the leukocytes to the top chamber. Migrated cells were detected in the lower chamber at the end of the experiment. Plots show one representative assay performed in triplicate out of at least three independent experiments. Error bars represent standard deviation. (**D**) Coomassie staining showing a representative purification of the rSgC used in the chemotaxis studies. **P<0.*005*; ***P<0.*0005*.

We addressed whether VZV rSgC interaction with chemokines had any functional relevance on chemokine activity by performing chemotaxis experiments using transwell devices. Addition of increasing concentrations of CXCL12-α resulted in migration of human leukemic CD4 T-cell line Jurkat, reaching a plateau at a chemokine concentration of approximately 15 nM (Fig 2A). Incubation of CXCL12-α with rSgC at a constant molar ratio (1:200, ck: rSgC) displaced the chemotactic curve towards lower chemokine concentrations, reaching the maximum number of migrated cells at a chemokine concentration of approximately 3 nM. The number of cells migrating at the peak of the chemotactic curve remained similar (Fig 2A). Thus, in the presence of rSgC the chemokine-induced migration of Jurkat T cells was enhanced at lower chemokine concentrations (1–3 nM), indicating that rSgC potentiates chemokine activity. Similar results were obtained with the human monocyte cell line MonoMac-1, indicating that this phenomenon was not cell type specific (Fig 2B). Due to the relevance of tonsillar leukocytes in the VZV infection cycle, we performed chemotaxis experiments using primary human leukocytes obtained from patients subjected to tonsillectomy. VZV rSgC also enhanced the CXCL12-α-dependent migration of human tonsillar leukocytes (Fig 2C).

### VZV rSgC binds to the cell surface through a specific interaction with GAGs

**Fig 3:**
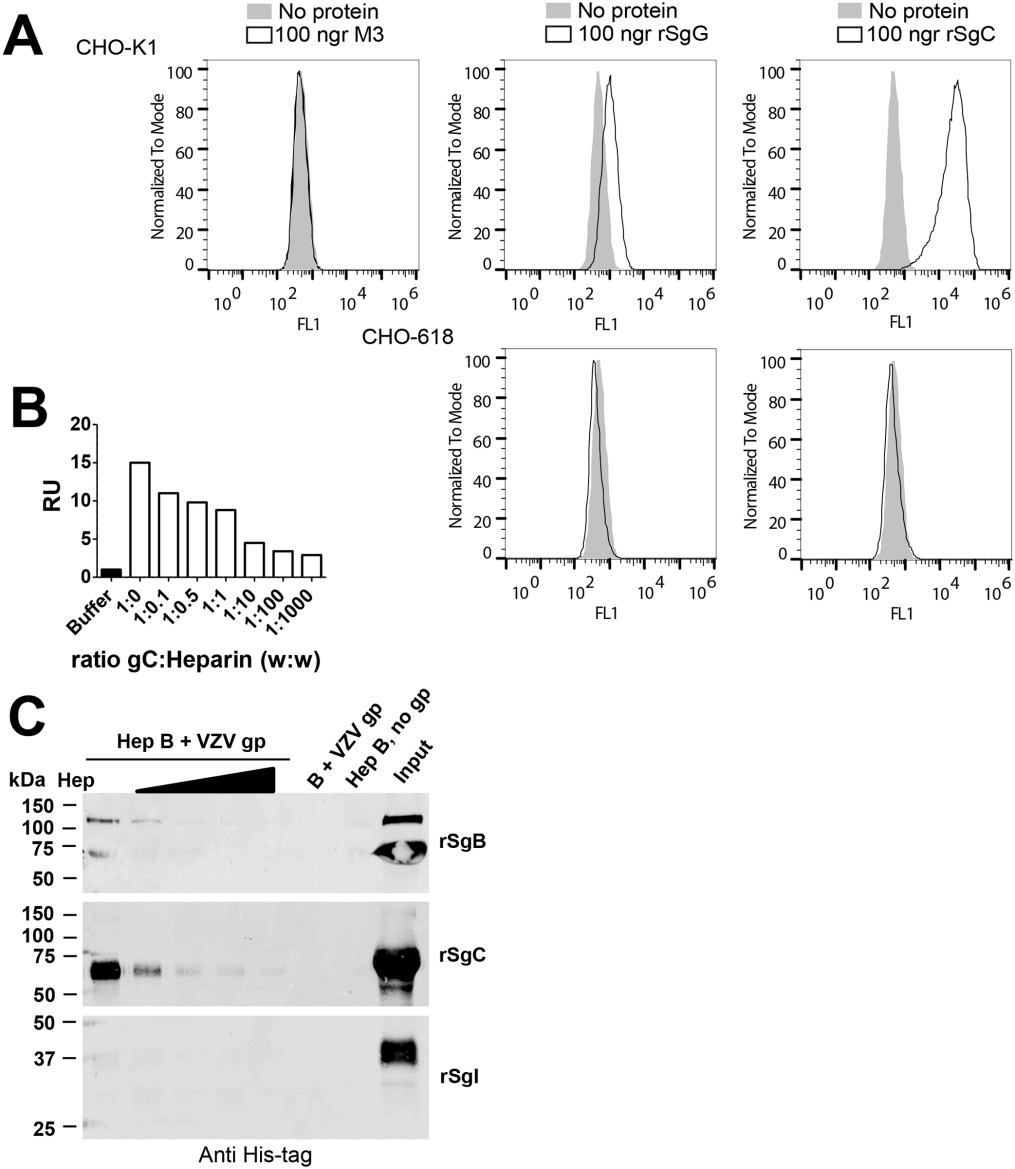
VZV rSgC interacts with the cell surface through a specific interaction with GAGs. (**A**) Histograms showing the interaction of MHV-68 M3 (left panel), HSV-2 rSgG (middle panels) and VZV rSgC (right panels) with CHO-K1 cells (upper panels) or CHO-618 cells (lower panels). CHO-K1 cells contain GAGs whereas CHO-618 cells are devoid of GAGs. Surface-bound proteins were detected by flow cytometry using an anti His-tag antibody. Light grey histograms represent the signal obtained when no recombinant protein was used. Empty histograms represent the signal obtained with 100 ng of purified recombinant protein. (**B**) Graph showing the number of resonance units (R.U.) obtained when rSgC (alone or in the presence of increasing concentrations of heparin) was injected over an SA chip containing immobilised heparin. The maximum R.U., recorded at 90 seconds, is shown. The signal obtained with buffer alone was subtracted from the signal obtained with the samples containing rSgC. The ratios of rSgC:heparin used are indicated in the X axis. (**C**) Western blots showing binding of VZV rSgB (top blot), VZV rSgC (middle blot) or VZV rSgI (bottom blot) to heparin beads. Bound proteins were detected by Western blotting using an anti His-tag antibody. Binding was competed with increasing amounts of soluble heparin (0.1, 0.5, 1 and 2 mg). The input, corresponding to 1/10 of the starting material, is shown in the right lane. One representative experiment out of at least three independent experiments is shown in **A-C**. Abbreviations: Hep, heparin; Hep B, heparin beads; gp, glycoprotein; rSg, recombinant soluble glycoprotein; kDa, kiloDaltons.

Several vCKBPs, but not all, bind to the plasma membrane through GAGs and this interaction seems to be important for their activity [31]. To our knowledge, so far it has not been formally proven that VZV gC binds GAGs. Previous work indicates that other proteins like glycoprotein B but not gC are responsible for initial interaction of VZV particles with GAGs [42]. VZV rSgC bound to the cell surface of Chinese hamster ovary cells (CHO-K1); but not to mutant CHO cells lacking GAG expression (CHO-618 cells) [48] (Fig 3A). As a positive control for GAG interaction we used HSV-2 recombinant SgG (rSgG) [49] and as a negative control M3 from MHV-68 [50]. The interaction of VZV rSgC with GAGs was confirmed by SPR using an SA chip containing biotinylated heparin (Fig 3B) and by pull-down assays using heparin beads (Fig 3C). VZV recombinant soluble glycoprotein B and I (rSgB and rSgI, respectively) were used as positive and negative controls for the interaction with heparin, respectively [51]. The interaction with heparin was specific since it could be competed with soluble heparin in the SPR and pull-down experiments, and rSgC did not bind to agarose beads lacking heparin (Fig 3B and 3C).

### Identification of VZV rSgC residues involved in chemokine and cell surface interaction

**Fig 4:**
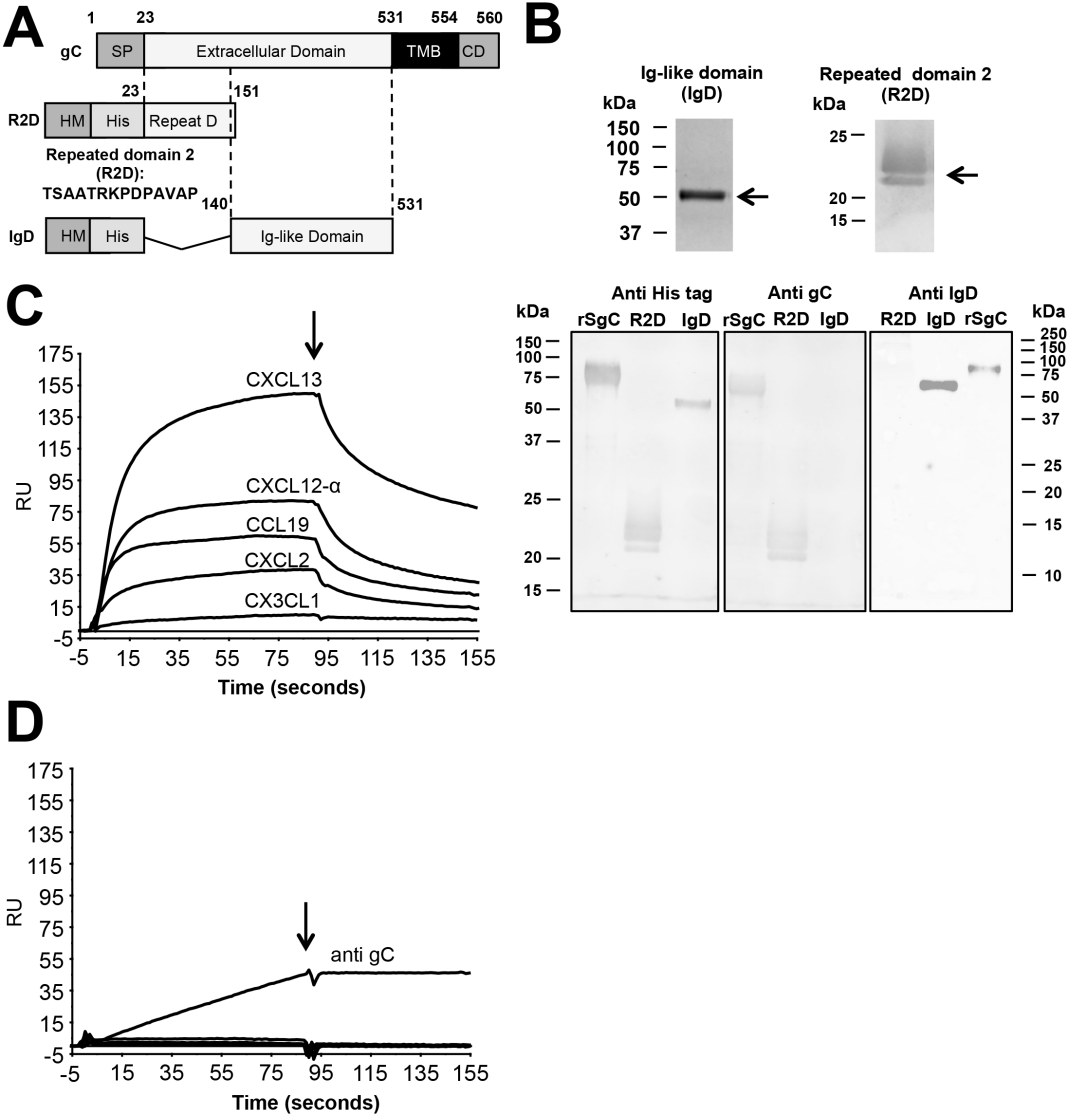
Identification of the rSgC binding domain responsible for interaction with chemokines. (**A**) Schematic representation of full-length gC protein (top construct) and deletion constructs containing either amino acids 23–151 (R2D, middle construct) or amino acids 140–531 (IgD, bottom construct). The numbers indicate amino acid positions within VZV gC Dumas strain. To improve secretion in insect cells the VZV gC signal peptide (SP) was substituted by that of the honey bee melittin (HM). The introduction of the N-terminal histidine tag (His) allowed purification of the proteins by affinity chromatography. (**B**) Purified proteins were detected by Coomassie staining (upper panels) or by Western blotting (bottom panels) using antibodies: anti His-tag (left panel), anti R2D (middle panel) and anti IgD (right panel). Left and middle blots were obtained following transfer from the same gel, whereas the right blot comes from an independent gel. (**C,D**) Sensorgrams showing the association and dissociation phases of the interaction between chemokines (CXCL2, CXCL12-α, CXCL13, CCL19 and the negative control CX3CL1 at 100 nM) and IgD (**C**) or R2D (**D**). The same chemokines were injected in the IgD and R2D chips. The arrow indicates the end of the chemokine injection. (**D**) An antibody targeted to gC was injected into the R2D chip at a concentration of 10 ng/μl. Abbreviations: RU, resonance units; kDa, kiloDaltons; TMB, transmembrane; CD, cytoplasmic domain.

VZV rSgC can be divided in two main regions according to its amino acid sequence. The N-terminal region (amino acids P23 to F151) contains a repeated sequence of fourteen amino acids (TSAASRKPDPAVAP) [52]. The number of repetitions varies among different strains [52]. Glycoprotein C from the Dumas strain, used in this study, contains seven and a half repetitions. The C-terminal region of the ectodomain (amino acids P140 to V531) contains two predicted immunoglobulin-like domains (according to Superfamily 1.75; Interproscan 5 and Phyre software) (Fig 4A). To determine the relevance of these two main regions in rSgC-chemokine interaction and modulation we generated two truncated rSgC proteins, one containing the repeated domain (amino acids P23 to F151) termed R2D and another one containing amino acids P140 to V531, including the predicted immunoglobulin-like domains, termed IgD (Fig 4A). Both constructs were expressed and purified using the same protocol as with the full-length rSgC, and therefore contain the honey bee melittin signal peptide and an N-terminal His-tag (Fig 4A). Purified recombinant proteins were detected by Coomassie staining and western blotting with an anti His-tag antibody. A previously generated anti-gC antibody [45] recognised the R2D, but not the IgD protein. To detect IgD we generated a new rabbit polyclonal antibody targeting purified recombinant IgD containing a C-terminal Twin-Streptavidin (Twin-Strep) tag [53] (IgD-Strep, S1 Fig). IgD-Strep was expressed in Schneider’s *Drosophila melanogaster* S2 cells and purified by affinity and size exclusion chromatography (S1 Fig). Both R2D and IgD were recognised by antibodies specific for each SgC region (Fig 4B).

Next we addressed which part of SgC binds chemokines by SPR. As shown in Fig 4C, IgD interacted with the same chemokines as rSgC and with similar KD (see also Table 2). IgD-Strep also interacted with chemokines (S1 Fig), confirming the relevance of this domain in the interaction using independent expression and purification systems. On the contrary, R2D did not interact with chemokines (Fig 4D). As a positive control for immobilisation of R2D in the Biacore chip we used a VZV gC-specific monoclonal antibody [45] (Fig 4D). Our results show that amino acids P140 to V531 of VZV rSgC are involved in the high affinity interaction with chemokines.

**Table 2:** Binding parameters of different chemokines to IgD. N.D: Not determined.

Chemokine	Binding	Ka (1/Ms)	Kd (1/s)	KD (M)	t(1/2)s
hCXCL12-α	Yes	1.26 x 10^7^	0.001601	1.27 x 10^−10^	393.50
hCXCL13	Yes	2.64 x 10^6^	0.001496	5.67 x 10^−10^	463.23
hCCL2	Yes	2.14 x 10^5^	0.001297	6.05 x 10^−9^	534.31
hCCL13	Yes	1.30 x 10^7^	0.003887	2.99 x 10^−10^	178.28
hCCL19	Yes	1.37 x 10^7^	0.001608	1.18 x 10^−10^	430.97
hCCL3	No	N.D.	N.D.	N.D.	N.D.
hCCL15	No	N.D.	N.D.	N.D.	N.D.

**Fig 5:**
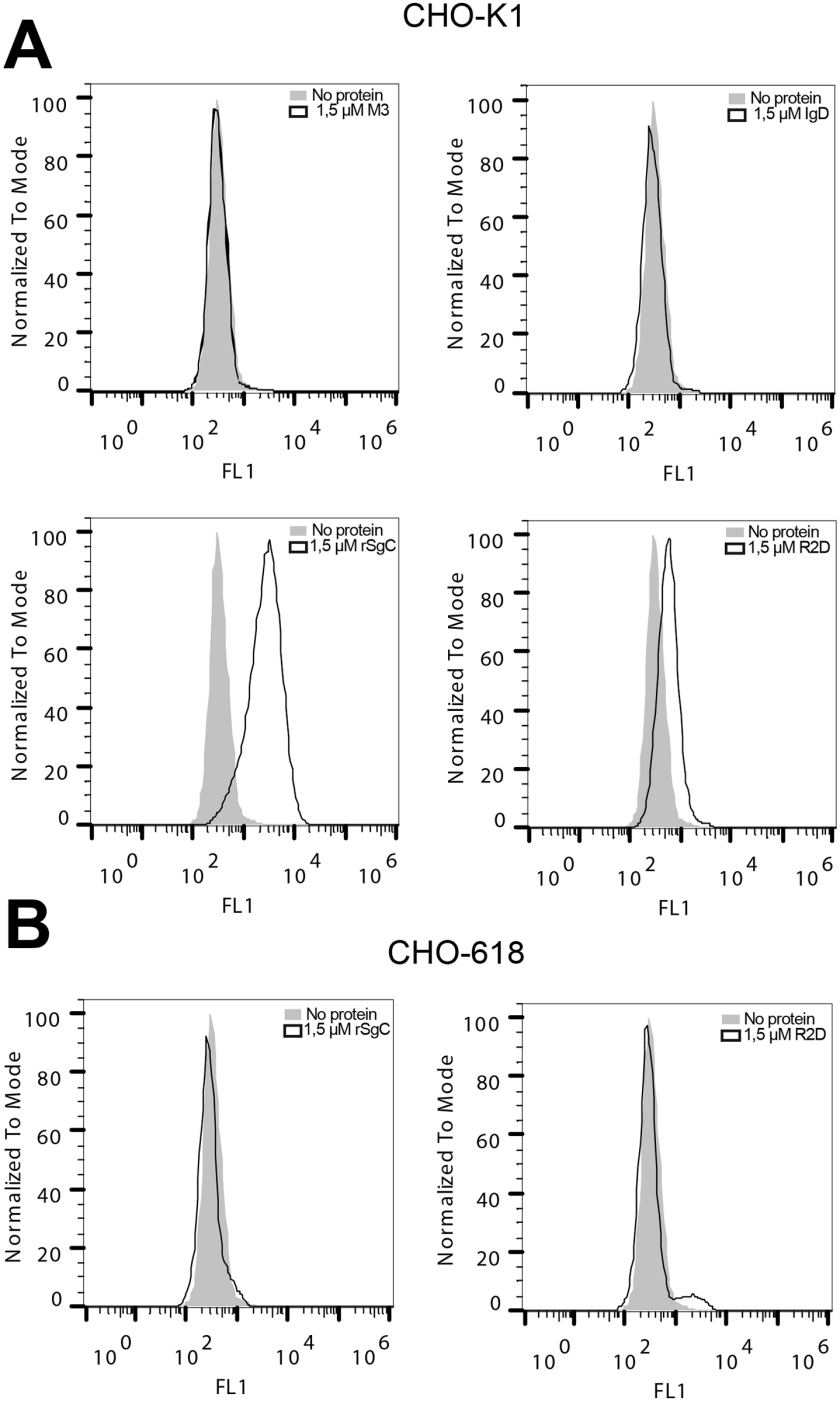
Characterization of the rSgC binding domain responsible for interaction with the cell surface. Histograms showing the interaction of purified recombinant M3, full-length rSgC, IgD and R2D with CHO-K1 cells (**A**) or CHO-618 cells (**B**). CHO-K1 cells contain GAGs whereas CHO-618 cells are devoid of GAGs. Bound proteins were detected by flow cytometry using an anti His-tag antibody. Light grey histograms represent the signal obtained when no recombinant protein was used. Empty histograms represent the signal obtained with recombinant protein. One representative experiment out of at least three independent experiments is shown.

We then performed cell-binding experiments to determine whether one of these two regions was responsible for the interaction with the cell surface. As shown in Fig 5A, the full-length rSgC and, to a lesser extent, R2D interacted with the cell surface of CHO-K1 cells. The interaction was dependent on the presence of GAGs since rSgC and R2D did not interact with the GAG-deficient CHO-618 cell line (Fig 5B). IgD did not bind to CHO cells, indicating that it does not interact with GAGs.

### Potentiation of chemokine activity *in vitro* does not require interaction of rSgC with the cell surface through GAGs

**Fig 6:**
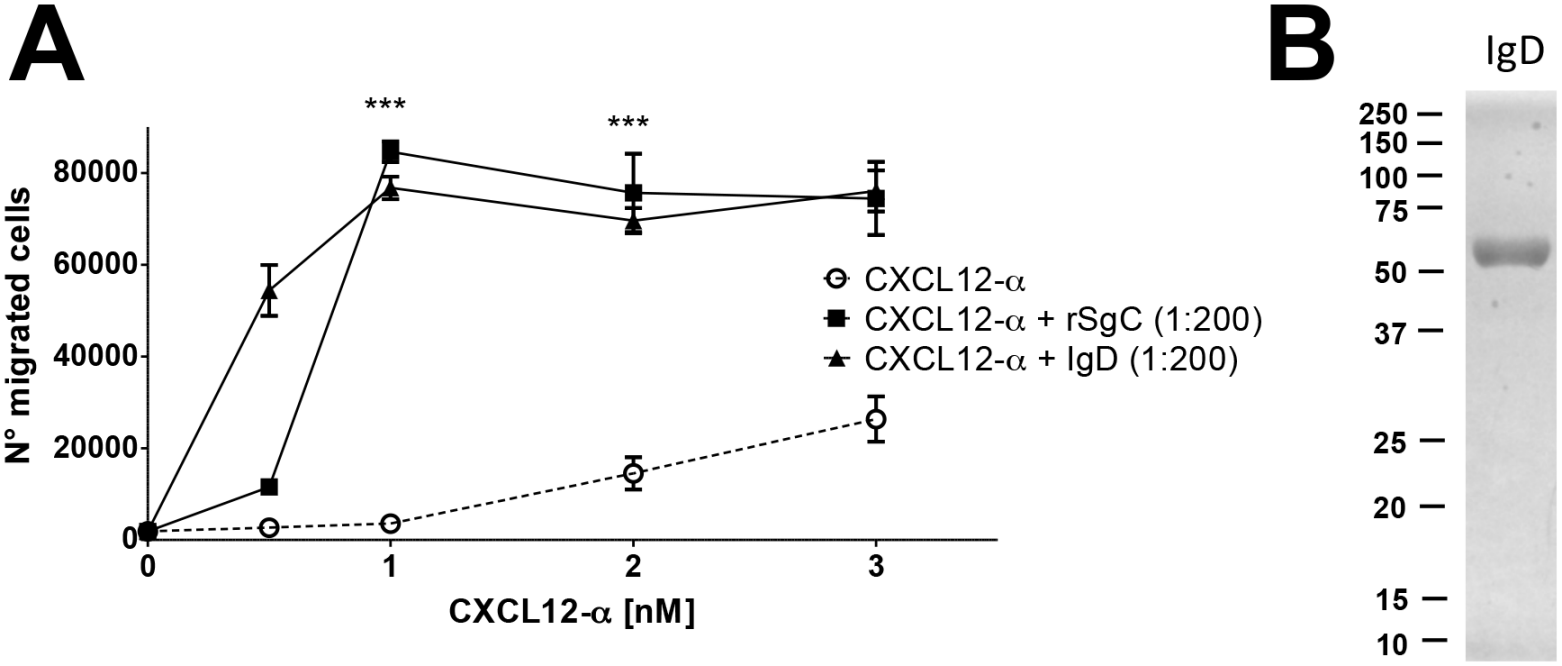
rSgC and IgD enhance chemokine-dependent migration. (**A**) Transwell experiment showing the effect of rSgC or IgD proteins on CXCL12-α-induced migration. A range of chemokine concentrations alone or together with 1:200 molar ratio of chemokine:rSgC or IgD was incubated in the bottom chamber of the transwell during 30 minutes at 37°C in a humidified incubator prior to the addition of Jurkat T cells to the top chamber. Migrated cells were detected in the bottom chamber. Plots show one representative assay performed in triplicate out of at least three independent experiments. Error bars represent standard deviation. (**B**) Coomassie staining showing a representative purification of the IgD protein used in the chemotaxis experiments. ***P<0.*0005*.

Analogous to HSV rSgG, VZV rSgC potentiates chemokine activity. Similarly, several host chemokines have been shown to enhance the chemotactic activity of other chemokines in a process referred to as chemokine synergism or cooperation [54]. Chemokines and HSV rSgG bind GAGs and this interaction appears to be relevant for the mechanism of action of both HSV rSgG and some synergistic chemokines [49, 55]. Therefore, we determined whether the interaction of VZV rSgC with the cell surface through GAGs was relevant for VZV rSgC activity. To this end we performed chemotaxis experiments with IgD, which bound chemokines with similar affinities as full-length rSgC (Fig 4C and Table 2), but did not interact with the cell surface through GAGs (Fig 5A). As shown in Fig 6, both IgD and rSgC potentiated chemokine activity to a similar extent. Similarly, IgD-Strep, purified by affinity and size exclusion chromatography, also enhanced chemokine activity as efficiently as IgD (S1 Fig). Thus, our results indicate that VZV rSgC interaction with the cell surface is not required for potentiation of chemokine activity *in vitro*.

### VZV rSgC enhancement of chemokine activity requires interaction with the chemokine and subsequent signalling through the chemokine receptor

**Fig 7:**
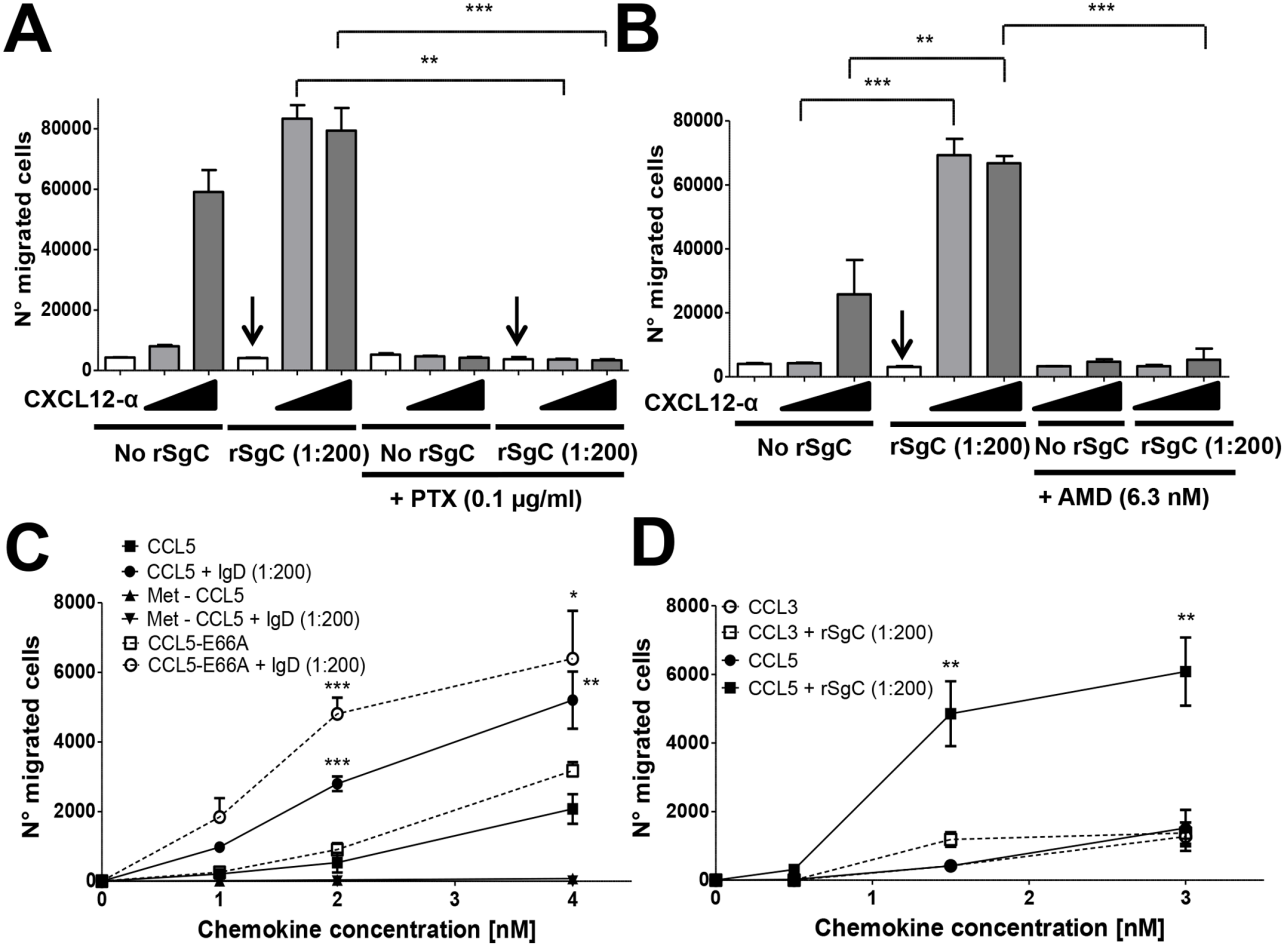
VZV rSgC enhancement of chemokine activity requires interaction with the chemokine and subsequent signalling through the chemokine receptor. Transwell experiment showing the effect of pertussis toxin (PTX) (**A**) or AMD3100 (**B**) on the chemotaxis of Jurkat T cells towards increasing concentrations of CXCL12-α alone or in the presence of 1:200 molar ratio of chemokine:rSgC. The arrows in (**A, B**) point to the condition with rSgC only, without chemokine. Transwell experiment showing the migration of THP-1 cells (**C, D**) towards increasing concentrations of wild type or mutated CCL5 (**C**) or CCL3 and CCL5 (**D**) alone or in the presence of 1:200 molar ratio of chemokine:IgD (**C**) or chemokine:rSgC (**D**). In all experiments the chemokine was incubated alone or together with VZV rSgC at 37°C in a humidified incubator in the bottom chamber of the transwell prior to the addition of the leukocytes to the top chamber. Migrated cells were detected in the lower chamber at the end of the experiment. Plots show one representative assay performed in triplicate out of at least three independent experiments. Error bars represent standard deviation. *P<0.*05*; **P<0.*005*; ***P<0.*0005*.

We next addressed whether VZV rSgC enhanced chemokine activity in the absence of chemokines, i.e., independent of chemokine-mediated receptor activation. We observed that the presence of the chemokine was required for rSgC-mediated enhancement since VZV rSgC alone did not induce migration (Fig 7A and 7B). Moreover, treatment of the sample with proteinase K (PKrSgC) abrogated rSgC activity, indicating that the effect was not due to the presence of a contaminating, non-proteinaceous compound such as lipopolysaccharide, in our preparation (S2A Fig). rSgC enhanced chemokine activity at chemokine:rSgC ratios lower than 1:200 (S2B and S2C Fig). VZV rSgC enhancing effect was blocked following addition of pertussis toxin (PTX) (Fig 7A), indicating that G protein coupling was required. Addition of AMD3100, an antagonist of CXCR4 [56], blocked rSgC activity showing that chemokine binding to its receptor and thereby signalling through the CXCL12 receptor are required (Fig 7B). These results were supported by the use of Met-CCL5, an antagonist of CCL5-mediated chemotaxis of primary human monocytes [57]. Pre-incubation of VZV IgD with Met-CCL5 did not result in chemotaxis, indicating again that chemokine activity is required for IgD potentiating effect. However, VZV IgD enhanced the activity of a non-aggregating CCL5, CCL5-E66A [58] (Fig 7C), which is fully active for chemotaxis *in vitro* [59], indicating that VZV IgD activity is independent of chemokine oligomerization *in vitro*. Lack of potentiation of Met-CCL5 was not due to lack of interaction with VZV IgD, since it interacted with both Met-CCL5 and CCL5-E66A similarly, as shown by SPR (S3 Fig). Finally, rSgC enhanced CCL5- but not CCL3-mediated chemotaxis of human monocytic THP-1 cells, a cell line migrating towards both chemokines (Fig 7D). Similar results were obtained using CXCL12-α, CCL3 and MonoMac-1 cells (S4 Fig). Since rSgC interacted with CXCL12-α and CCL5 but not with CCL3 (Fig 1C), these results show that interaction of rSgC with the chemokine is required for rSgC activity. Overall, these results show that VZV rSgC does not induce chemotaxis on its own but enhances the activity of chemokines through a mechanism that requires chemokine interaction and signalling through the chemokine receptor.

### Cell-free VZV enhances chemokine activity and this effect is partially dependent on gC

**Fig 8:**
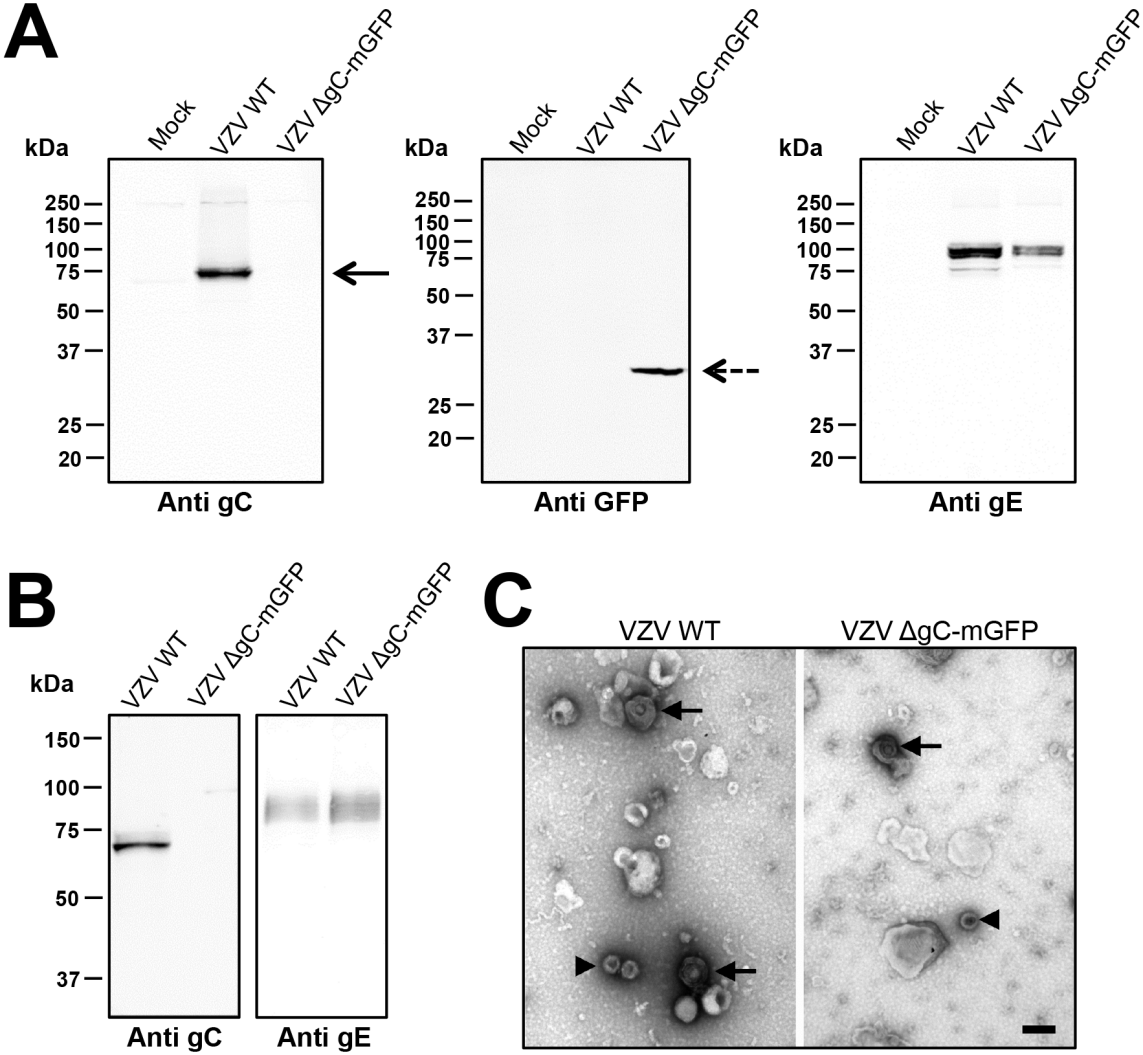
Characterization of recombinant VZV lacking gC expression. (**A**) Western blots showing expression of gC (left panel), mGFP (middle panel) and gE (right panel) in cell lysates of ARPE-19 cells infected with pOka-WT or pOka-ΔgC-mGFP (VZV WT and VZV ΔgC-mGFP, respectively). The gE and gC panels show the same blot subjected to sequential antibody staining following stripping of the membrane. (**B**) Western blots showing presence of gC (left panel) or gE (right panel) in cell-free VZV produced from MeWo cells infected with pOka-WT or pOka-ΔgC-mGFP (VZV WT and VZV ΔgC-mGFP, respectively). The two panels show the same blot subjected to sequential antibody staining following stripping of the membrane. (**C**) Representative electron micrograph of cell-free pOka-WT or pOka-ΔgC-mGFP (VZV WT and VZV ΔgC-mGFP, respectively) subjected to negative staining. Arrows point to enveloped virions, arrowheads to capsids. The magnification bar represents 200 nm.

**Fig 9:**
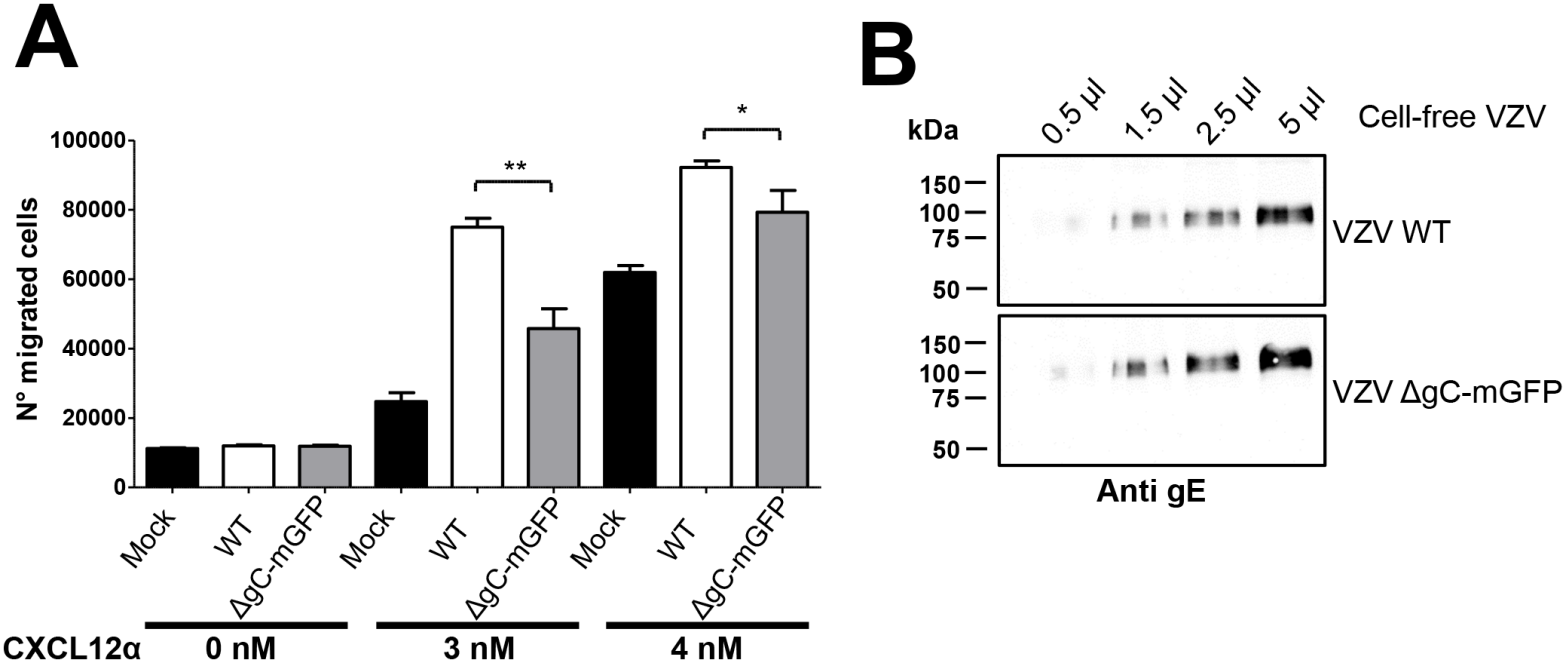
VZV enhances chemokine-dependent migration of T cells and gC is partially responsible for this effect. (**A**) Graph showing the number of Jurkat T cells migrating towards 2.8 μl of pOka-WT or pOka-ΔgC-mGFP (VZV WT and VZV ΔgC-mGFP, respectively) cell-free VZV alone or together with chemokine in a transwell assay. Similar amounts of cell-free VZV were used based on gE expression. Migrated cells were detected in the lower chamber at the end of the experiment. Plots show one representative assay performed in triplicate out of at least three independent experiments. Error bars represent standard deviation. (**B**) Western blot showing similar level of gE in pOka-WT or pOka-ΔgC-mGFP (VZV WT and VZV ΔgC-mGFP, respectively) cell-free VZV used in the chemotactic assay. *P<0.*05*; **P<0.*005*.

We next addressed whether gC modulates chemokine activity during VZV infection. To this end we generated a recombinant VZV, based on VZV strain pOka, expressing monomeric green fluorescent protein (mGFP) instead of gC (pOka-ΔgC-mGFP) using the *en passant* mutagenesis [60]. Recombinant viruses were characterized by western blotting (Fig 8), and sequenced to ensure the lack of non-desired mutations. Deletion of gC did not impair replication in the human retinal epithelial cells ARPE-19 (S5 Fig), analogous to previous studies [39, 42]. We generated and analysed cell-free VZV by western blotting and negative staining (Fig 8). Both wt and pOka-ΔgC-mGFP had similar levels of gE expression and viral particle morphology. We performed chemotactic assays using comparable virus amounts based on the level of gE protein (Fig 9). Pre-incubation of the CXCL12-α with parental cell-free pOka enhanced migration of Jurkat cells towards the chemokine. The enhancement in migration by cell-free VZV required the presence of the chemokine, because the virus alone did not induce chemotaxis, similar to rSgC. This is the first description of such a phenomenon for VZV. The use of cell-free pOka-ΔgC-mGFP resulted in approximately 20–50% reduced enhancement of chemokine activity depending on the experiment and the amount of chemokine used (Fig 9), indicating that gC plays a role in VZV mediated enhancement, and that in addition other unknown factors are involved.

## Discussion

Following primary infection of epithelial cells in the respiratory tract, VZV infects and replicates in dendritic and T cells allowing systemic dissemination of the virus [11, 12, 61]. The cellular and viral factors responsible for hijacking leukocytes are not fully known. We have identified a VZV protein, gC, that enhances chemokine-dependent leukocyte migration. Since VZV gC is expressed with true late kinetics [62, 63], when infectious viral particles are produced, gC may facilitate the recruitment and infection of leukocytes and optimize virus dissemination within the infected individual.

We showed that cell-free VZV enhanced chemokine activity and that this activity is partially dependent on gC. VZV is strictly cell associated *in vitro* and has been found in a cell-free form in suprabasal keratinocytes and blister fluid *in vivo* [64]. To our knowledge, secretion of cell-free virus by VZV-infected cells in the respiratory mucosa has not been described. Importantly, the gC enhancing effect was also observed with primary human tonsillar leukocytes. We hypothesise that VZV gC enhances their migration to the initial site of infection. The role of VZV gC *in vivo* is not completely understood, in part due to the difficulties of studying VZV infection in animal models. We do not know at which step of the VZV lytic cycle gC activity may play a role but our data indicate that it is also active at the level of the viral particles. Virion-associated gG from HSV also enhances chemokine-mediated migration [65]. VZV gC is relevant for the infection of human skin cells in a humanized severe combined immunodeficiency mouse model [40]. Lack of gC seems to result in diminished spread through the epidermis towards the dermis, which could be due to impairment of infection of polarized epithelial cells [40]. Chemokine interaction with its receptor leads to the activation of signalling proteins resulting in disassembly of tight junctions and actin remodelling among other processes [66–68]. One could envisage that an increased GPCR signalling involving gC-chemokine interaction with the cognate chemokine receptor may facilitate the early steps of viral infection of polarised epithelial cells. Finally, gC activity could be relevant for VZV-associated pathology since chemokines can modify neuronal activity and induce pain [37, 38]. Further experiments are required to determine the role of gC-mediated chemokine enhancement *in vivo*.

Chemokines orchestrate the migration and activity of leukocytes and play key roles in the interplay between the innate and adaptive immune response. We show here that VZV rSgC binds a broad range of CXC and CC chemokines with nanomolar affinity and therefore acts as a novel vCKBP. vCKBP have been described in poxviruses [69–77] and in herpesviruses [33, 46, 47, 78–80]. Notably, this report constitutes the first description of a vCKBP in VZV. This is particularly relevant since VZV does not contain *US4*, the gene encoding the vCKBP expressed by several alphaherpesviruses [33, 80–83]. Moreover, VZV rSgC is novel since it binds a large number of chemokines specifically from the CXC and CC subfamilies and, especially, because it enhances their activity, something only previously observed for HSV gG [34]. However, HSV-1 and HSV-2 rSgG bind a reduced number of CXC and CC chemokines. The wider interacting range of gC probably reflects the relevance of leukocytes during VZV primary infection. The fact that only human alphaherpesviruses express a vCKBP with the ability to enhance chemokine activity may reflect yet unknown evolutionary requirements. Enhancement of chemokine function is similar to the activity of human synergistic or cooperative chemokines, which enhance the chemotactic properties of other chemokines [54]. Three different mechanisms that can explain chemokine cooperation between different chemokines have been postulated. One involves the convergence of signalling pathways triggered by independent interactions between the chemokines and their respective receptors [84–86]. Another requires GAG competition between cooperative chemokines resulting in higher levels of free chemokine that interact with its receptor [55]. The third one postulates that the formation of heteromeric complexes between the chemokines is required for synergy [87–89]. In the case of vCKBP that enhance chemokine activity, e.g., HSV rSgG and VZV rSgC, convergence signalling does not seem to be responsible for the mechanism of action since the vCKBPs alone do not induce chemotaxis or even signalling as shown here and previously [34]. Moreover, addition of AMD3100 and the lack of enhancement when the Met-CCL5 mutant is used indicate that the activity requires binding of the chemokine to its receptor and subsequent signalling. GAG interaction could play a relevant role since both HSV rSgG and VZV rSgC interact with the plasma membrane through a high affinity interaction with GAGs, as shown here and previously [49]. However, interaction with GAGs, for VZV rSgC at least, does not seem to be relevant for enhancing chemokine activity *in vitro*, since an rSgC construct lacking GAG binding (VZV IgD) enhances chemokine activity as efficiently as the full-length rSgC. However, *in vitro* transwell experiments may not provide sufficient information regarding the role of GAGs in chemotaxis. *In vivo*, lack of GAG interaction could result in loss of gC activity as shown for an interferon binding protein expressed by vaccinia virus [90]. Similar to some human synergistic chemokines, the mechanism of action of VZV rSgC involves chemokine interaction and signalling through the chemokine receptor.

Overall, we identified gC as a protein potentially involved in the recruitment of leukocytes by the highly infectious, neurotropic human pathogen VZV. Our data clearly show that VZV gC enhances leukocyte migration through modulation of chemokine activity, a phenomenon that could facilitate VZV infection of leukocytes and subsequent spread. Furthermore, since chemokines can induce pain [91], enhancement of their activity could play a role in VZV associated pathology. Further investigations are required to understand the relevance of VZV gC activity in the context of VZV spread and pathogenesis *in vivo*.

## Materials and methods

### Ethical statement

The human tonsil specimens were obtained in the context of surgical interventions (ethical approval number 1916–2013), the experiments were performed according to the approved guidelines.

### Cells and viruses

Hi-5 adherent insect cells were grown in Grace insect medium (Sigma-Aldrich, Germany) supplemented with 10% FBS. Hi-5 and Schneider’s *Drosophila melanogaster* Line 2 (S2) cells suspension insect cells were grown in Insect Xpress medium (Lonza) without serum. For transfection with the construct P-068 encoding for IgD-Strep (see below) S2 cells were grown in Schneider’s Drosophila medium (Gibco by Life Technologies) supplemented with 10% FBS. Stably transfected S2 cells were grown in Insect-Xpress medium supplemented with 8 μg/mL puromycin (Invivogen). All insect cells were grown at 28°C. Jurkat T cells E6.1 and THP-1 (both provided by Martin Messerle, Hannover Medical School, Germany) and Macrophage-monocyte 1 (MonoMac-1) cells (a gift from Antonio Alcami, Centro de Biología Molecular Severo Ochoa, Madrid, Spain) were grown in RPMI 1640 (Gibco) supplemented with 10% FBS. CHO-K1 and CHO-pgsB-618 [48] (CHO-618, provided by Antonio Alcami) were grown in DMEM-F12 (Gibco) 1:1 medium containing 10% FBS. Human melanoma MeWo cells were purchased from American Type Culture Collection (ATCC-HTB-65) and grown in DMEM containing 10% FBS. Human lung fibroblast MRC-5 cells and human retinal epithelial ARPE-19 cells were a gift from Martin Messerle. MRC-5 were grown in DMEM containing 10% FBS and ARPE-19 were grown in DMEM/Nutrient mixture F-12 Ham medium (Sigma) containing 8% FBS. All mammalian cell lines were cultured at 37°C, 5% CO_2_ in a humidified incubator. Tonsillar leukocytes were prepared as previously described [92].

VZV Dumas strain [36] (a gift of Andrew Davison, University of Glasgow, U.K.) was maintained in MRC-5 cells. Bacterial artificial chromosome (BAC)-derived VZV pOka strain was a gift from Nikolaus Osterrieder (Freie Universität Berlin, Germany). The virus was reconstituted in MeWo cells using Lipofectamin 2000 (ThermoFisher, see below).

### Cloning, expression and purification of recombinant proteins

The coding sequences for the ectodomains of VZV SgC, SgB and SgI without their putative signal peptides and SgC truncated constructs (IgD and R2D) were cloned into pFastBacMel [34]. The genbank accession number for the Dumas strain is NC_001348.1. The gene IDs are 1487660, 1487662 and 1487689 for gC, gB and gI, respectively. The resulting DNA constructs contain the honey bee melittin signal peptide followed by an N-Terminal His-tag and the VZV glycoprotein ectodomain sequence. VZV rSgC, rSgB and rSgI ectodomains were amplified from DNA obtained from MRC-5 cells infected with VZV Dumas strain [36]. The following primers were used: 5’-TATGGCGCCCCCACACCCGTAAGTATAACT-3’ and 5’-TATTTAGGTACCTTAAACGGAAAATGTAGTGGC-3’ containing *Nar*I and *Kpn*I sites, respectively, for rSgC; 5’-TATTATGGCGCCGTTGTGTCGGTCTCTCCAAGC-3’ and 5’-TATTTACGTACGTTACCCAAATGGGTTAGATAAAAA-3’ containing *Nar*I and *Sph*I sites, respectively, for rSgB and 5’-TATTATGGCGCCATCTTCAAGGGCGACCAC-3’ and 5’-AATTATCGTACGTTATTCTGGAGGATCATTAAGGGA-3’ containing *Nar*I and *Sph*I sites, respectively, for rSgI. Truncated rSgC constructs, IgD and R2D, were amplified from pFastBacMel-rSgC plasmid using the primers 5’-TATGGCGCCCCCACACCCGTAAGTATAACT-3’ and 5’-TATTTAGGTACCAAAAGGTGGTTGTGAATG-3’ containing *Nar*I and *Kpn*I sites, respectively, for R2D and 5’-TATGGCGCCCCCGCAGCCAACAACCAA-3’ and 5’-TATTTAGGTACCTTAAACGGAAAATGTAGTGGC-3’ containing *Nar*I and *Kpn*I sites, respectively, for IgD. All plasmids were sequenced to ensure the lack of mutations. Recombinant baculoviruses were obtained following transformation of the respective pFastBacMel plasmid DNA into DH10Bac cells and subsequent transfection of the recombinant bacmids into Hi-5 adherent insect cells using Lipofectamine 2000 (Invitrogen). The supernatant of infected adherent Hi-5 cells was collected at 72 hours post infection to perform heparin-pulldown experiments. Recombinant proteins (rSgC, IgD and R2D) were purified from the supernatant of infected Hi-5 suspension cells 84 hours post infection with recombinant baculovirus by affinity chromatography using Nickel beads (Qiagen), as before [81]. To express large amounts of IgD the VZV IgD sequence (amino acids 140–531, Dumas strain) was amplified using oligonucleotides 5’-TATTTAACTAGTAACGGAAAATGTAGTGGC-3’ and 5’-TATAGATCTCCCGCAGCCAACACCCAA-3’ containing *Spe*I and *Bgl*II, respectively, and cloned into a *Drosophila melanogaster* expression plasmid [53]. The resulting construct (P-068) contains the *Drosophila* immunoglobulin binding chaperone protein signal peptide (BiP), IgD and a C-terminal segment coding for a specific proteolytic cleavage site, followed by a Twin-Strep-Tag, secreting soluble IgD-Strep into the supernatant. The plasmid was sequenced to ensure the lack of undesired mutations. This plasmid was co-transfected into Schneider’s *Drosophila melanogaster* S2 cells together with a dominant selectable marker encoding a puromycin resistance gene to generate a stable cell line as described before [93]. For transfection S2 cells were in Schneider’s Drosophila medium (Gibco by Life Technologies) supplemented with 10% FBS. The transfection reagent used was Effectene Enhancer (QIAGEN). The expression of the recombinant IgD-Strep was induced by adding 4 μM cadmium chloride to cells growing at a density of 6–9 x 10^6^ cells/ml. IgD-Strep was purified from the supernatant of S2 cells by affinity chromatography using a Strep-Tactin Superflow high capacity column (IBA GmbH, Germany). The affinity-purified protein was subjected to size exclusion chromatography using a HiLoad 26/600 Superdex 200 pg (GE Healthcare Life Sciences). The resulting IgD-Strep protein was used to generate a rabbit polyclonal antibody targeting the VZV gC IgD.

The purity and concentration of all purified proteins was determined by comparing Coomassie R250 (TH. Geyer) stained bands of recombinant protein with a BSA standard curve loaded in the same gel. Purified recombinant proteins were detected by SDS-PAGE and Western blotting using mouse monoclonal antibodies diluted in PBS Tween containing 3% milk targeted to either the His-tag (Qiagen) or the recombinant protein. The anti gC antibody has been previously characterized [45]. The generation of the anti IgD-Strep antibody is described below. Fluorescently labelled antibody was used as secondary antibody and the signal was detected with Licor (Odissey). Recombinant, purified M3 and rSgG2 were provided by Antonio Alcami.

### Generation of a polyclonal antibody to the VZV IgD construct

Purified recombinant IgD-Strep was used to inject two rabbits at Davids Biotechnology (Germany). IgD anti-serum was obtained at day 63 post-immunization.

### Chemokines

Recombinant human chemokines used in SPR experiments (CXCL1, CXCL2, CXCL3, CXCL4, CXCL5, CXCL6, CXCL7, CXCL8, CXCL9, CXCL10, CXCL11, CXCL12-α, CXCL12-β, CXCL13, CXCL14, CXCL16, CCL1, CCL2, CCL3, CCL4, CCL5, CCL7, CCL8, CCL11, CCL14 (66 aa), CCL14 (72 aa), CCL15, CCL16, CCL17, CCL18, CCL19, CCL20, CCL21, CCL22, CCL23, CCL24, CCL25, CCL26, CCL27, CCL28, CX3CL1 and XCL1), or in chemotaxis experiments (CXCL12-α, CCL2, CCL3, CCL5 and CCL7) were from Peprotech and were reconstituted in 1x PBS with 0.1% BSA as a carrier protein.

### Determination of rSgC-chemokine binding specificity and affinity constants using SPR technology

To determine whether VZV rSgC bound human chemokines and to calculate the affinity constants of such interactions we performed SPR experiments with a BIAcore X-100 biosensor (GE Healthcare). All chips were purchased from GE Healthcare. In all experiments with rSgC, IgD, R2D and IgD-Strep, the recombinant proteins were amine-coupled in acetate buffer pH 5.0. Binding screenings and experiments to calculate the association and dissociation constants (*k*_*a*_ and *k*_*d*_, respectively) of the interactions were performed using CM4 chips. Screening experiments with IgD-Strep were carried out with a CM5 chip containing 2864 immobilised R.U. The number of R.U. coupled in the CM4 chips were 774.8 for rSgC, 611 for IgD and 245.7 for R2D (*R*_*max*_ < 100 RU). The differences in the number of immobilised units are due to the different molecular weights of the recombinant proteins (72.5 kDa for rSgC, 55 kDa for IgD and 22.5 kDa for R2D). To detect immobilised R2D, a mouse monoclonal anti gC antibody [45] was injected at a concentration of 10 ng/μL during 90 s. Recombinant chemokines (Peprotech) reconstituted in PBS containing 0.1% BSA were injected at 100 nM in HBS-EP buffer (10 mM HEPES, 150 mM, NaCl, 3 mM EDTA, 0.005% (v/v) surfactant P20, pH 7.4) at a flow rate of 30 μl/min for screening experiments (90 s contact time, 60 s dissociation). Multi-cycle kinetics experiments were carried out by injecting different concentrations of the chemokine at 30 μl/min (180 s contact time, 600 s dissociation). In all cases the chip surface was regenerated after each chemokine injection with 10 mM glycine–HCl pH 2.0. All BIAcore sensorgrams were analysed with the Biacore X100 Evaluation Software. Bulk refractive index changes were removed by subtracting the reference flow cell responses, and the average response of a blank injection was subtracted from all analyte sensorgrams to remove systematic artefacts. Kinetic data were globally fitted to a 1:1 Langmuir model. When required, NSB Reducer (GE Healthcare) was used to reduce non-specific binding to the dextran matrix of the chips.

### Chemotaxis assays

Different chemokine concentrations, alone or in combination with purified recombinant VZV rSgC, R2D, IgD or cell-free VZV, were placed in the lower compartment of a 96-well ChemoTx System plate (Neuro Probe Inc., MD, USA) in RPMI 1640, with the exception of the cell-free virus, which was present in PBS. Similar amounts of cell-free VZV controlled by viral titre and protein expression were used. As controls, R-1 medium or recombinant protein alone in R-1 medium, or cell-free virus alone in PBS, were used. The cells (Jurkat, MonoMac-1, THP-1 or human tonsillar leukocytes, at a concentration of 5x10^6^/ml) were separated from the lower chamber by a 3 μm (for MonoMac-1 cells and tonsillar leukocytes) or 5 μm filter (Jurkat cells and THP-1). The cells were incubated at 37°C for 2–3 h in a humidified incubator with 5% CO_2_. The number of migrated cells in the lower chamber was determined by staining with 5 μl of CellTiter 96 aqueous one solution cell proliferation assay (Promega, WI, USA) during 1.5 h at 37°C, with 5% CO_2_, measuring absorbance at 490 nm and comparing the absorbance values with those of a standard curve obtained using known cell numbers. The number of migrated THP-1 and tonsillar leukocytes was determined using a light microscope as they did not react to CellTiter 96 aqueous one solution cell proliferation assay. To determine the effect of PTX (Tocris) on cell migration, we incubated 0.1 μg/ml of PTX with Jurkat cells overnight prior to the chemotaxis experiment. To address whether CXCL12-α receptor was involved in rSgC mechanism of action, Jurkat T cells were incubated with 6.3 nM AMD3100 in R-1 medium during 15 minutes at room temperature, prior to the chemotaxis experiments. The cells were tested without washing the drug to avoid receptor reactivation [94]. To address the effect of possible non-proteinaceous elements in our protein preparation, we incubated rSgC with proteinase K at a concentration of 60 nM at 44°C for 30 min followed by heat-inactivation (95°C during 20 min).

### Cell binding assays

CHO-K1 and CHO-618 cells detached with PBS-EDTA were incubated with 1.5 μM of purified rSgC, IgD, R2D, M3 and rSgG2 for 20 min at 4°C. Following three washing steps with PBS at 4°C, the cells were incubated with 10 μg/ml of anti His-tag antibody (QIAGEN) followed by incubation with 4 μg/ml of alexa fluor 488-conjugated anti mouse antibody (Thermofisher scientific). Three washing steps with PBS at 4°C were carried out after each antibody incubation step. The data were collected on a FACS FC500 (Beckman and coulter) and analysed using FlowJo.

### Determination of rSgC-GAG binding specificity using SPR technology

Biotinylated heparin was coupled on a Biacore SA chip (252 RU) using the SA-biotin capture method. Briefly, immobilization buffer (1M NaCl and 50 mM NaOH) was injected followed by injection of biotinylated heparin in H_2_O and addition of 50 mM NaOH, 1M NaCl, 50% isopropanol. To determine the specificity of the interaction between rSgC and heparin, rSgC was injected at a flow rate of 10 μl/min (90 s contact time, 60 s dissociation) at a concentration of 1.8 ng/μl in presence of different amounts of heparin (Sigma-Aldrich, Germany) at a rSgC:heparin ratio (w:w) of 1:0; 1:0.1; 1:0.5; 1:1; 1:10; 1:100; 1:1000.

### Heparin pull-down assay

Supernatants from Hi-5 adherent cells containing recombinant proteins were incubated with heparin-sepharose beads (Sigma-Aldrich, Germany) for 1.5 h at 4°C. Recombinant proteins were detected with the anti His-tag antibody by Western blotting prior to the pulldown to ensure that similar amounts of all recombinant proteins were used in the assay. The binding of recombinant proteins to the heparin-beads was competed by adding 0.1 to 2 mg of soluble heparin (Sigma-Aldrich, Germany) to the binding reaction. As negative control, agarose beads lacking heparin were used (Sigma-Aldrich, Germany). The beads were washed three times with PBS and the proteins were eluted with denaturalizing loading buffer for SDS-PAGE. The presence of the recombinant proteins was detected by Western blotting using the anti His-tag antibody.

### Generation of recombinant VZV lacking ORF14 expression

Recombinant VZV lacking gC expression was generated using the BAC technology and *en-passant* mutagenesis as described previously [60, 95]. The constructed mutant is based on the infectious BAC of the pOka strain previously generated [96]. To abrogate gC expression ORF14 was replaced by a monomeric GFP (mGFP)-cassette (BAC-pOka-ΔgC-mGFP). The BAC was mutated in ORF14 by insertion of a mGFP-Kanamycin resistance (KanR) cassette in which an excisable KanR gene disrupts the mGFP ORF. The KanR is flanked by a duplicated fragment of mGFP sequence and I-*Sce*I restriction sites, which allows subsequent excision of KanR and the seamless repair of the mGFP ORF by Red recombination in E.coli strain GS1783 [60]. The cassettes were amplified by PCR with the plasmid pEP-mGFP-in [97] as template and using the primers “For”: 5’-TTTATTTAAGGGGAGCGTGGATGTGTCAATAAAAACCAGGATGGTGAGCAAGGGCGAGGA-3’ and “Rev”: 5’-AATAAAATGATATACACAGACGCGTTTGGTTGGTTTCTGTTTACTTGTACAGCTCGTCCATG-3’ to replace the gC with the mGFP gene in the BAC. Successful recombination was confirmed by restriction analysis (*Nhe*I + *Xba*I double digest) and Illumina sequencing (MiSeq) of the viral genomes.

To reconstitute infectious recombinant viruses MeWo cells were transfected with fresh BAC DNA using Lipofectamin 2000 (ThermoFisher) in a 6-well plate (10 μl Lipofect + 10–50 μl of BAC-VZV DNA), resulting in the recombinant virus pOka-ΔgC-mGFP used in this manuscript. Lipofect-DNA complexes were produced in OptiMEM and dripped onto subconfluent (~80%) MeWo cells. After 24 h medium was changed and cells were incubated with maintenance splits of cells every week until formation of syncytia. The expression of mGFP, gC and gE was assessed by western blotting using a monoclonal commercial antibody for GFP (Clontech) and previously described mouse monoclonal antibodies for gC and gE [45].

### VZV replication kinetics

Subconfluent ARPE-19 cells (approx. 80% confluency) were infected with VZV by adding infected MeWo cells (equivalent to 100 plaque forming units (pfu)/well). Cultures were then incubated for 6 days and at each time point respective cultures were 1x rinsed with PBS and then trypsinized with 100 μl trypsin-EDTA solution (GIBCO) for 5 min. 400 μl medium (DMEM+20% FCS) was then added and the cells were washed off the well. This cell suspension was then combined with 500 μl medium + DMSO (DMEM + 20% FCS + 20% DMSO) and the cells were frozen using an isopropanol chamber. All samples were then analysed in parallel in a plaque assay by infecting subconfluent ARPE-19 cells with 250 μl of cell suspension in 5-fold dilution series. Cells were incubated for 4 days and plaques were counted using a light microscope.

### Generation of cell-free VZV

10 p150 dishes containing MeWo cells at 90–100% confluency were infected with cell-associated VZV (pOka, pOka-ΔgC-mGFP) and harvested in PBS when 30–50% of cells showed cytopathic effect. The cells were sonicated (3 times during 15 s with a 15 s interval) on ice with a Bandelin Sonorex RK100 sonicator and centrifuged during 15 min at 1,000 x g and 4°C. The supernatant was transferred to a new tube and mixed with ice cold Lenti-X concentrator (Clontech) at a Lenti-X:supernatant ratio of 1:4. The solution was incubated during 2 hours at 4°C followed by 45 min centrifugation at 1,500 x *g* and 4°C. The supernatant was collected and centrifuged through a 10% (w/v) Nycodenz (Axis-Shield PoC) cushion in PBS at 34766.4 *g* and 4°C. The resulting pellet was resuspended in PBS, aliquoted and stored at -80°C.

### Negative staining of cell-free VZV

For negative staining cell-free VZV preparations were adsorbed onto carbon and Formvar-film coated 400 mesh copper grids (Stork Veco). After washing with PBS and distilled water, preparations were negative stained using 2% (w/v) uranyl acetate, and analyzed with a Morgani (FEI, Einthoven, Netherlands) transmission electron microscope at 80 kV.

### Statistical analysis

The significant value (*P* value) was calculated by performing unpaired Student T-test using GraphPad Prism.

## Supporting information

S1 Fig Expression and purification of recombinant IgD-Strep to generate a rabbit polyclonal antibody. (A) Schematic representation of full-length VZV gC (top) and the derived construct to express soluble VZV IgD containing a Twin-Streptavidin tag (IgD-Strep, bottom) in S2 insect cells. Numbers indicate amino acid positions within VZV gC Dumas strain. The VZV gC signal peptide (SP) was substituted by that of the Drosophila immunoglobulin binding chaperone protein (BiP) to improve secretion in S2 insect cells. A Twin-Streptavidin (Strep) tag was introduced at the C-terminus to facilitate purification of IgD by affinity chromatography. (B) Purified IgD-Strep was detected by Coomassie staining. (C) Sensorgram showing association and dissociation phases of the interaction between IgD-Strep immobilised in a CM5 chip and selected chemokines injected at a concentration of 100 nM. The arrow indicates the end of the chemokine injection. Positive (CXCL13, CXCL12-α, CXCL2 and CCL19) and negative (CX3CL1) interactions are shown. (D) Chemotaxis of Jurkat cells towards increasing concentrations of CXCL12-α alone or in the presence of a 1:200 molar ratio of chemokine:IgD or chemokine:IgD-Strep. The chemokine alone or together with IgD or IgD-Strep was incubated in the bottom chamber of the transwell at 37°C in a humidified incubator prior to the addition of the leukocytes to the top chamber. Migrated cells were detected in the lower chamber at the end of the experiment. Plots show one representative assay performed in triplicate out of at least three independent experiments. Error bars represent standard deviation. Abbreviations: RU, resonance units. kDa, kiloDaltons.***P<0.*0005*.
(TIF)

S2 Fig Recombinant VZV rSgC preparation lacks non-proteinacious contaminants potentiating chemokine activity. Chemotaxis of Jurkat T cells towards 1 nM (**A**) or 2 nM (**B**) of CXCL12-α alone or in the presence of a 1:33 (**A, B**), 1:66 or 1:270 (**A**) molar ratio of chemokine:rSgC. 1:270 molar ratio of chemokine:proteinase K-treated VZV rSgC (PKrSgC) was used as control (**A**). (**C**) Chemotaxis of THP-1 cells towards 3 nM of CCL5 alone or in the presence of a 1:15 molar ratio of chemokine:rSgC. The chemokine alone or together with VZV rSgC was incubated in the bottom chamber of the transwell at 37°C in a humidified incubator prior to the addition of the leukocytes to the top chamber. Migrated cells were detected in the lower chamber at the end of the experiment. Plots show one representative assay performed in triplicate out of at least three independent experiments. Error bars represent standard deviation. *P<0.*05*; **P<0.*005*; ***P<0.*0005*.
(TIF)

S3 Fig VZV IgD binds CCL5 mutants. Sensorgram showing the association and dissociation phases of the interaction between IgD and two CCL5 mutants, Met-CCL5 and CCL5-E66A, injected at a concentration of 100 nM. The arrow indicates the end of the chemokine injection. Abbreviations: RU, resonance units.
(TIF)

S4 Fig rSgC enhancement activity requires interaction with the chemokine. Chemotaxis of MonoMac-1 cells towards increasing concentrations of CXCL12-α or CCL3 alone or in the presence of a 1:200 molar ratio of chemokine:rSgC. The chemokine was incubated with or without VZV rSgC at 37°C in a humidified incubator prior to the addition of the leukocytes to the top chamber. Migrated cells were detected in the lower chamber at the end of the experiment. Plots show one representative assay performed in triplicate out of two independent experiments. Error bars represent standard deviation. *P<0.*05*; **P<0.*005*; ***P<0.*0005*.
(TIF)

S5 Fig Lack of gC expression does not hinder VZV replication kinetics. Graph showing the replication kinetics of pOka-WT and pOka-ΔgC-mGFP in ARPE-19 cells. To measure replication kinetics, ARPE-19 cells were infected with MeWo-associated virus and the cells were collected at different days post infection. These ARPE-19 cells were later added on naïve ARPE-19 cells and the number of plaque forming units per ml (PFU/ml) was determined at 72 hours post infection.
(TIF)
