# Supplementary figures and images for: Varicella zoster virus glycoprotein C increases chemokine-mediated leukocyte migration
Source: PLoS Pathog. 2017 May 25;13(5):e1006346. doi: 10.1371/journal.ppat.1006346 (PMC5444840; doi:10.1371/journal.ppat.1006346)

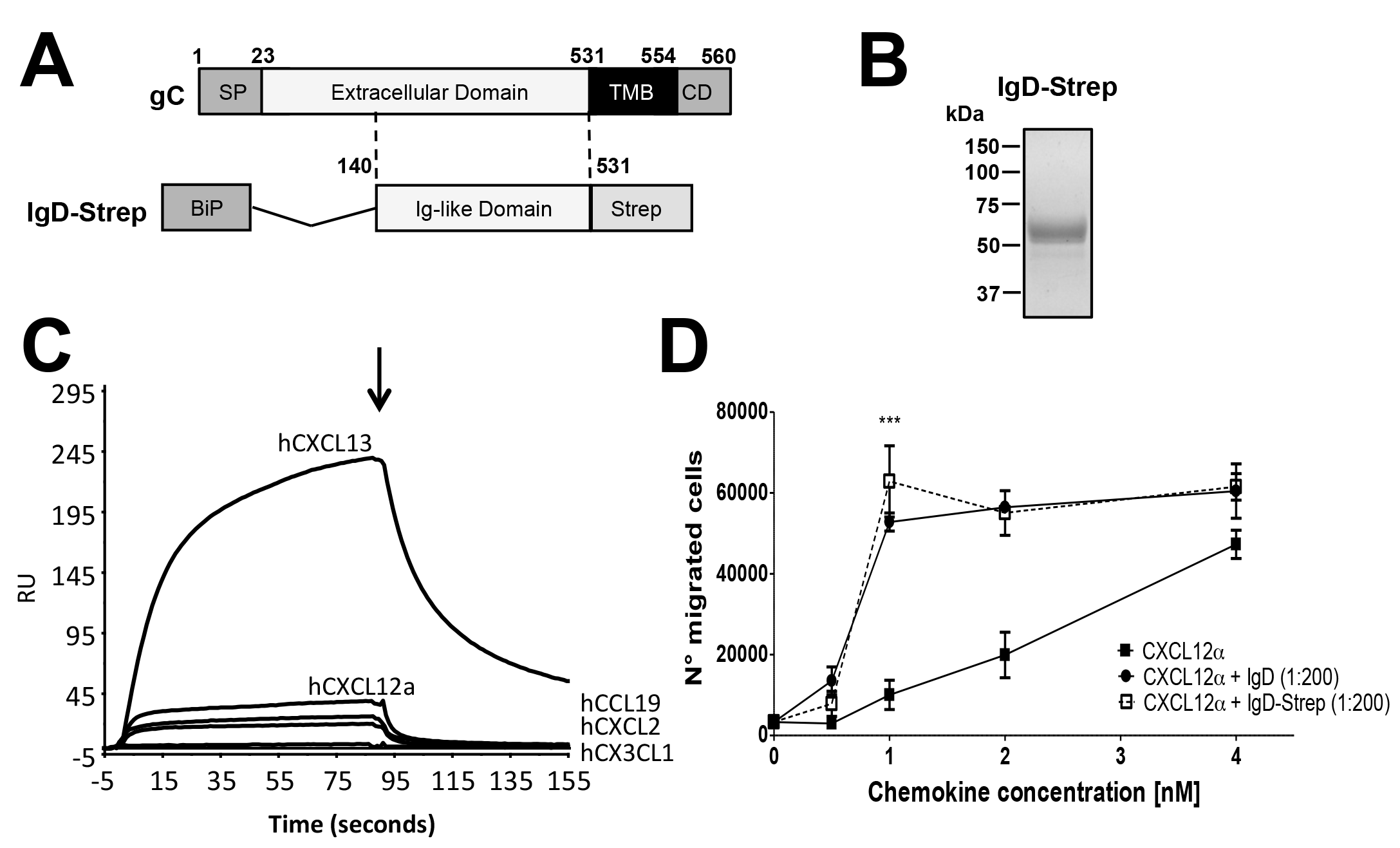

Supplement: S1 Fig — (A) Schematic representation of full-length VZV gC (top) and the derived construct to express soluble VZV IgD containing a Twin-Streptavidin tag (IgD-Strep, bottom) in S2 insect cells. Numbers indicate amino acid positions within VZV gC Dumas strain. The VZV gC signal peptide (SP) was substituted by that of the Drosophila immunoglobulin binding chaperone protein (BiP) to improve secretion in S2 insect cells. A Twin-Streptavidin (Strep) tag was introduced at the C-terminus to facilitate purification of IgD by affinity chromatography. (B) Purified IgD-Strep was detected by Coomassie staining. (C) Sensorgram showing association and dissociation phases of the interaction between IgD-Strep immobilised in a CM5 chip and selected chemokines injected at a concentration of 100 nM. The arrow indicates the end of the chemokine injection. Positive (CXCL13, CXCL12-α, CXCL2 and CCL19) and negative (CX3CL1) interactions are shown. (D) Chemotaxis of Jurkat cells towards increasing concentrations of CXCL12-α alone or in the presence of a 1:200 molar ratio of chemokine:IgD or chemokine:IgD-Strep. The chemokine alone or together with IgD or IgD-Strep was incubated in the bottom chamber of the transwell at 37°C in a humidified incubator prior to the addition of the leukocytes to the top chamber. Migrated cells were detected in the lower chamber at the end of the experiment. Plots show one representative assay performed in triplicate out of at least three independent experiments. Error bars represent standard deviation. Abbreviations: RU, resonance units. kDa, kiloDaltons.***P<0.0005. (TIF) [file ppat.1006346.s001.tif]

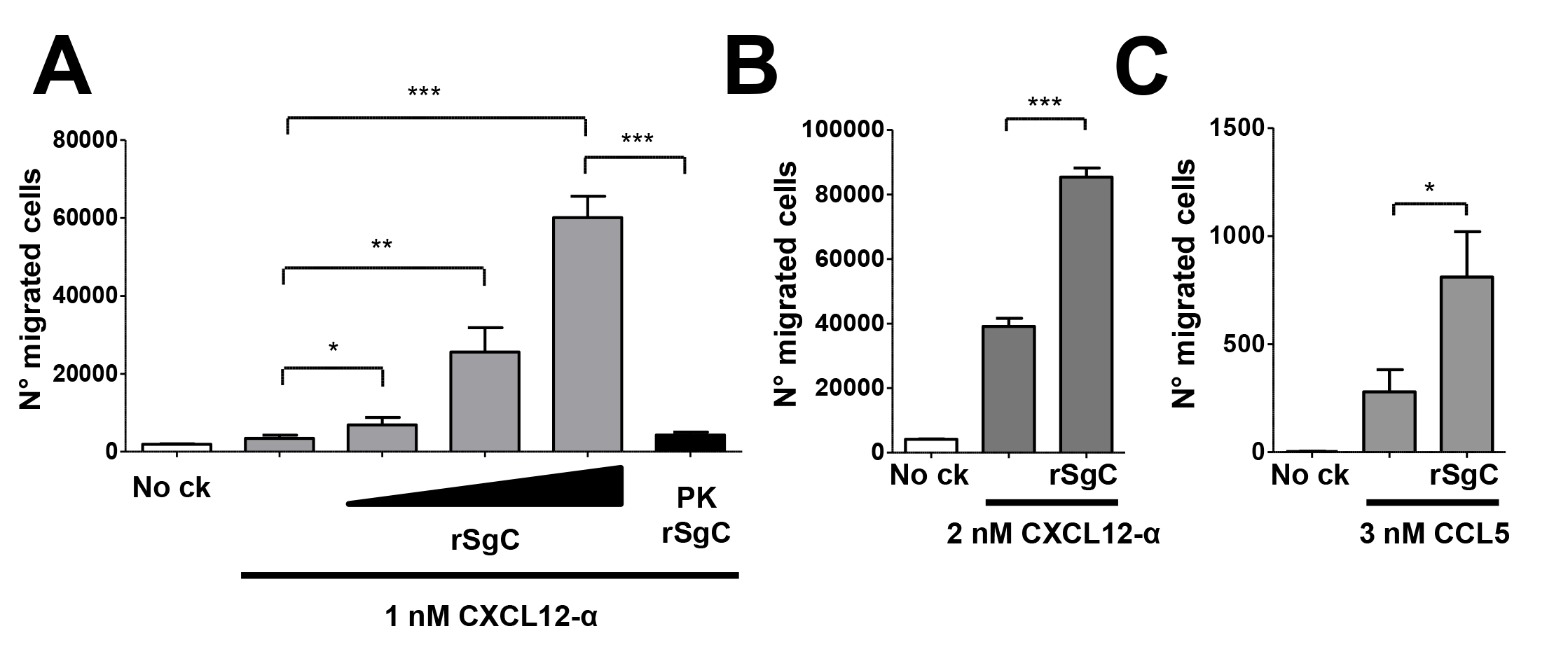

Supplement: S2 Fig — Chemotaxis of Jurkat T cells towards 1 nM (A) or 2 nM (B) of CXCL12-α alone or in the presence of a 1:33 (A, B), 1:66 or 1:270 (A) molar ratio of chemokine:rSgC. 1:270 molar ratio of chemokine:proteinase K-treated VZV rSgC (PKrSgC) was used as control (A). (C) Chemotaxis of THP-1 cells towards 3 nM of CCL5 alone or in the presence of a 1:15 molar ratio of chemokine:rSgC. The chemokine alone or together with VZV rSgC was incubated in the bottom chamber of the transwell at 37°C in a humidified incubator prior to the addition of the leukocytes to the top chamber. Migrated cells were detected in the lower chamber at the end of the experiment. Plots show one representative assay performed in triplicate out of at least three independent experiments. Error bars represent standard deviation. *P<0.05; **P<0.005; ***P<0.0005. (TIF) [file ppat.1006346.s002.tif]

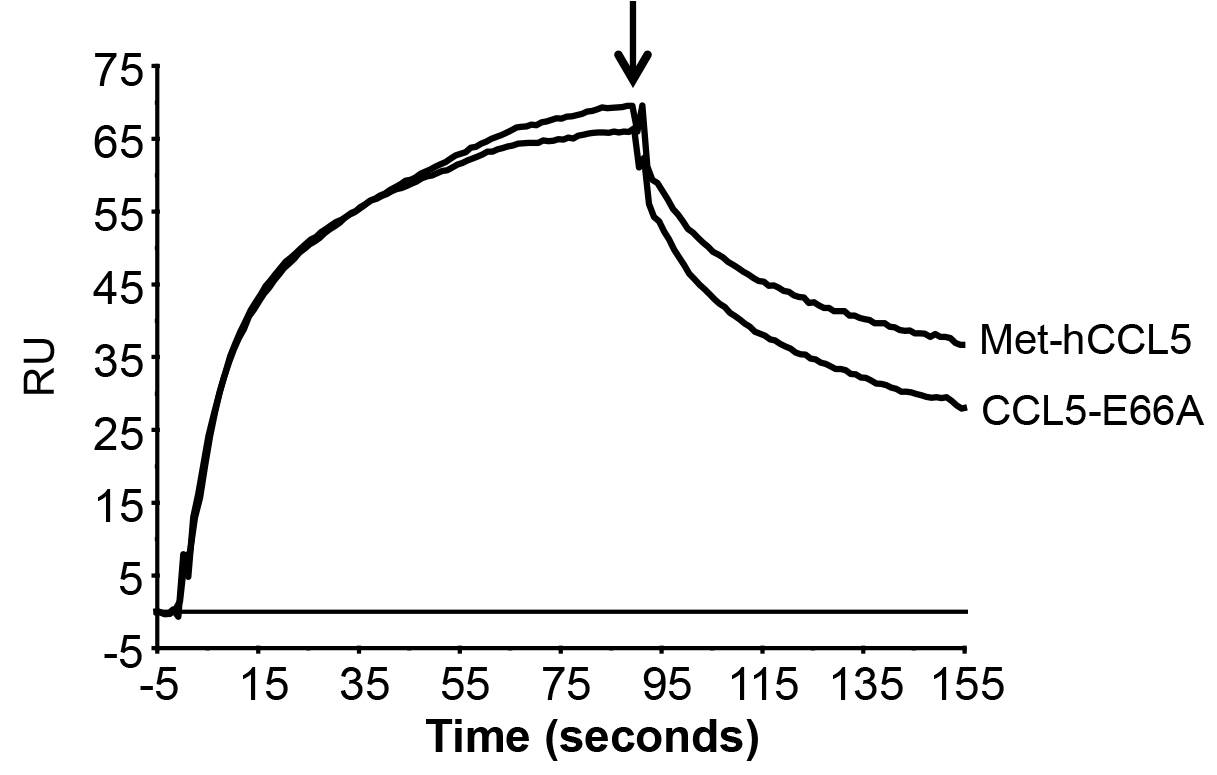

Supplement: S3 Fig — Sensorgram showing the association and dissociation phases of the interaction between IgD and two CCL5 mutants, Met-CCL5 and CCL5-E66A, injected at a concentration of 100 nM. The arrow indicates the end of the chemokine injection. Abbreviations: RU, resonance units. (TIF) [file ppat.1006346.s003.tif]

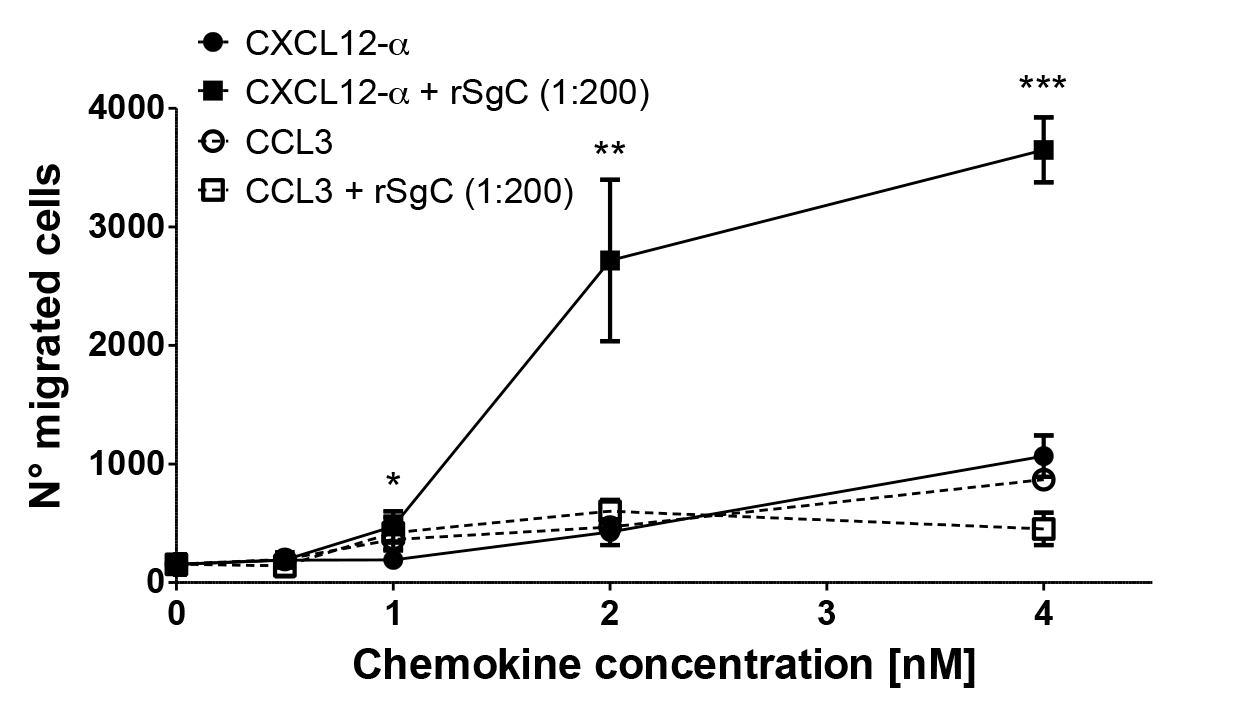

Supplement: S4 Fig — Chemotaxis of MonoMac-1 cells towards increasing concentrations of CXCL12-α or CCL3 alone or in the presence of a 1:200 molar ratio of chemokine:rSgC. The chemokine was incubated with or without VZV rSgC at 37°C in a humidified incubator prior to the addition of the leukocytes to the top chamber. Migrated cells were detected in the lower chamber at the end of the experiment. Plots show one representative assay performed in triplicate out of two independent experiments. Error bars represent standard deviation. *P<0.05; **P<0.005; ***P<0.0005. (TIF) [file ppat.1006346.s004.tif]

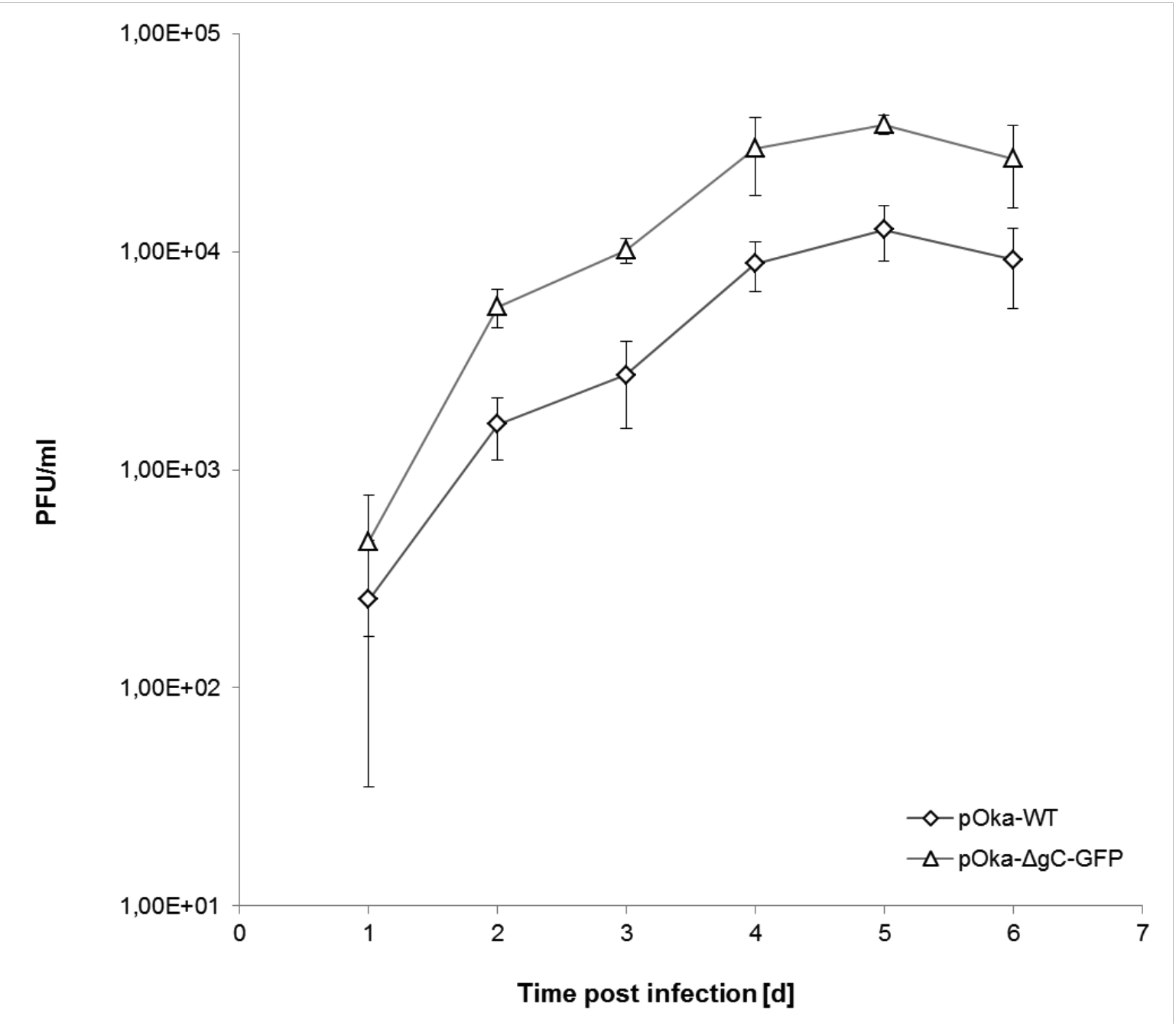

Supplement: S5 Fig — Graph showing the replication kinetics of pOka-WT and pOka-ΔgC-mGFP in ARPE-19 cells. To measure replication kinetics, ARPE-19 cells were infected with MeWo-associated virus and the cells were collected at different days post infection. These ARPE-19 cells were later added on naïve ARPE-19 cells and the number of plaque forming units per ml (PFU/ml) was determined at 72 hours post infection. (TIF) [file ppat.1006346.s005.tif]
